# Local brain volume reductions in patients with non-lesional epilepsy on 7T MRI

**DOI:** 10.1007/s00234-025-03843-3

**Published:** 2025-11-18

**Authors:** Johannes Schwarzer, Eya Khadhraoui, Eric Einspänner, Olga Kukhlenko, Daniel Behme, Lars Büntjen, Friedhelm C. Schmitt, Sebastian Johannes Müller

**Affiliations:** 1 Clinic for Neuroradiology, Otto-Von-Guericke-University Magdeburg, Magdeburg, Germany; 2 DZNE Magdeburg, Magdeburg, Germany; 3 Clinic for Neurology, Otto-Von-Guericke-University Magdeburg, Magdeburg, Germany; 4 Stimulate Research Campus Magdeburg, Magdeburg, Germany; 5 Clinic for Neurosurgery, Otto-Von-Guericke-University Magdeburg, Magdeburg, Germany

**Keywords:** FastSurfer, FreeSurfer, Epilepsy, Non-lesional epilepsy, Hippocampal region

## Abstract

**Objective:**

In patients with non-lesional epilepsy, MR imaging detects no structural or functional abnormalities. The aim of this study was to determine whether subtle local reductions or increases in brain volume, undetectable to the human eye, can indicate an epileptic focus directly or indirectly. To address this, we performed brain volumetry using 7T MRI.

**Methods:**

We evaluated 7T MRI in patients with non-lesional epilepsy as part of a retrospective study and a healthy control cohort from another prospective study. FastSurfer segmentations were performed using T1 MPRAGE. Additionally, we also performed volumetry of the hippocampal subfields, the thalamic nuclei and the brainstem. We created a control group matched for age and gender distribution.

**Results:**

7T segmentation as described above was possible in 14 patients with epilepsy and 27 participants of a control cohort. We detected a significant volume loss in the ipsilateral central lateral nucleus of thalamus, as well as a significant increase in the presubiculum body and the ipsilateral and contralateral entorhinal and medial orbitofrontal cortices.

**Conclusion:**

High-resolution 7T MRI-based volumetric analysis in patients with non-lesional epilepsy revealed significant atrophy in brain regions commonly implicated in epileptogenesis. These structures exhibited strong sensitivity and specificity, highlighting the potential of volumetry as a diagnostic tool in the absence of visible lesions. Validation in larger, independent cohorts is required to confirm these findings and assess clinical applicability.

**Supplementary Information:**

The online version contains supplementary material available at 10.1007/s00234-025-03843-3.

## Introduction

Today, MRI plays a crucial role in the etiological classification of epileptic symptoms. Reductions in brain volume play a less important role in the diagnosis, but they can also provide evidence of structural defects that can be both a cause and a consequence of epilepsy as a network disease [1].The outcome of epilepsy surgery (seizure freedom) is significantly better in patients which harbor lesions than in patients with non-lesional epilepsy (NLE) [2].

FastSurfer [3] is a standard tool for the assessment of brain atrophies especially in dementia diagnostics [4]. Since brain atrophy can often be detected in epilepsy, it might provide indirect clues to the seizure origin [5].

In 3 T studies, no significant atrophies have been identified in non-lesional epilepsy, except for a slight lateralization in patients with a long disease history [6].

Since 7 T MRI has improved lesion detection [7], question arises, firstly, whether it can reveal regional volume alterations in these patients that help to better understand the pathomechanisms, and secondly, whether it is suitable for use as a diagnostic criterion.

To further investigate this, we analyzed the brain volumes of patients with NLE using FastSurfer as part of a retrospective 7 T MRI study. Since the hippocampus also plays an important role, we also performed a hippocampal subfield segmentation.

## Methods

### Study design

This study is a retrospective analysis and was ethically approved by the institutional review board and adhered to the 2013 Declaration of Helsinki. The institutional review board waived the requirement for informed consent because of the retrospective nature of the study. The patients had given their consent for a secondary evaluation of their MRI data. All methods were performed in accordance with relevant guidelines and regulations.

The control group was obtained from participants of an earlier study aimed at generating normative data at 7T. The participants had given their consent for the secondary use of their data. The ethics committee approved this use in our study.

## Participant population

Seventeen patients with confirmed 3T-NLE were included in the study. The patients are a subgroup of the study by Kukhlenko et al. [8]. The original study included 20 individuals who underwent both 3 T and 7 T scans, all showing semiological features and ictal EEG findings consistent with non-lesional epilepsy on 3 T MRI.

The control group was generated from a different study (same scanner, same sequence) from healthy individuals without any known neurological disorders. We selected the participants to match the epilepsy group in terms of age and sex distribution (mean and standard deviation). Subgroups were not additionally matched for gender or age, since GLM analyses confirmed that neither Gender nor Age had a significant effect across regions.

## MRI protocol and technical details

The 3D (TR 2500 ms, TE 2 ms) T1-MPRAGE with a voxel-size of 0.7 mm x 0.7 mm x 0.7 mm was performed on MAGNETOM Terra 7 T (Siemens Healthineers AG, Werner-von-Siemens-Str. 1, D-80333 Munich, Germany).

## Pre-processing

Because of severe inhomogeneities, we had to perform an external bias correction and a skull-stripping algorithm performed in a python script.

Bias field correction is a crucial pre-processing step as it affects qualitative and quantitative image analysis. We used the N4 algorithm [9] to estimate the bias field from the image itself. The N4-based bias field correction was performed with a spline order of 3, control point grid size of 4 × 4 × 4, FWHM parameter of 0.15 and a convergence threshold of 0.00001. Subsequently, we performed skull-stripping using SynthStrip [10].

Additionally, we de-activated the included bias correction of FastSurfer [11].

To assess the effect of prior skull-stripping, we conducted an additional GLM analysis that included N4 bias correction but omitted external skull-stripping.

**Fig. 1:**
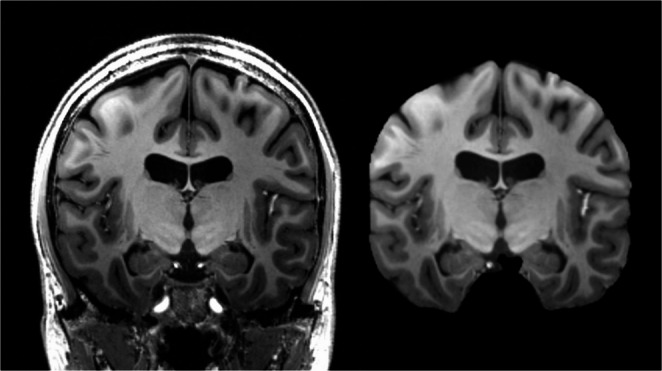
Coronary slice of the hippocampal region before (left) and after (right) n4 bias correction and skull stripping

Figure 1 demonstrates an example of T1-weighted images on 7 T MRI before and after correction.

## Volumetric assessment

We ran FastSurfer (Version 2.3.0) on T1-MPRAGE data of 7 T MRI, and calculated Desikan-Killiany-Tourville (DKT) [12] atlas volumes. Additionally, we measured subvolumes of hippocampus and amygdala using the FreeSurfer script “HippocampalSubfieldsAndNucleiOfAmygdala” (FreeSurfer Version 7.4.1).

### Statistical analysis

We used R-Studio (Version 2024.04.2) for statistical programming and histogram/image construction (additional packages: pROC, ggplot2).

For further analysis, we normalized all volumetric values by dividing them by the whole brain volume. We checked for normal distribution of the values with Shapiro-Wilk test [13]. The comparison of the groups (NLE patients and Healthy Control) and individual subgroups (left-sided and right-sided EEG focus) were performed with a Mann-Whitney-U test [14]. Significance level was set to 5%. Additionally we used Bonferroni’s correction [15].

To further test the robustness of the results obtained with the Mann-Whitney U test, we conducted an additional analysis using a Gaussian general linear model (GLM) implemented in RStudio.

Furthermore, to evaluate the diagnostic capabilities of the significant structures, we performed a receiver operating characteristic (ROC) analysis for group classification and for estimating the focus hemisphere.

## Results

### Participants

FastSurfer segmentation failed in one of 17 epilepsy patients due to movement artifacts. We excluded two of the remaining 16 epilepsy patients, because we detected a possible focal cortical dysplasia according to Wang et al. [16] and a tiny temporal meningocele with a related small area of microgyria on 7 T MRI. Finally, seven patients with mainly left-sided as well as seven patients with right-sided epilepsy were included.

We had to exclude the five oldest individuals of the control group (*n* = 32) in order to match average age and gender distribution in the epilepsy group.

**Fig. 2:**
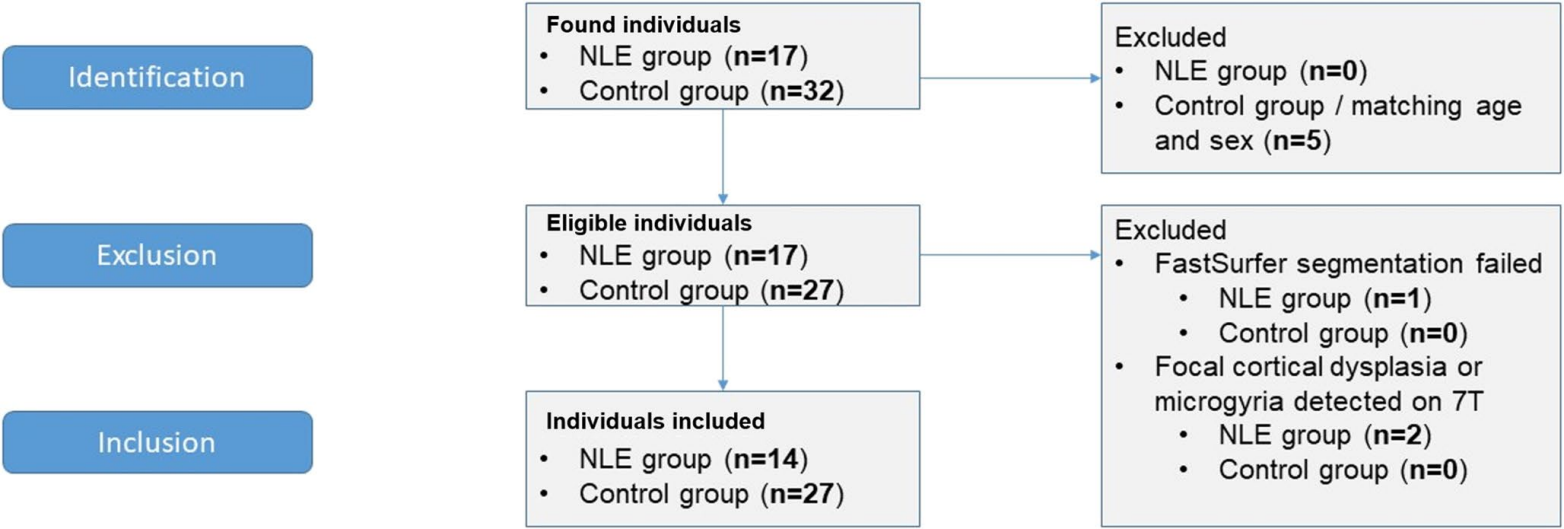
Inclusion flow chart. *Legend*: NLE – non-lesional epilepsy

Figure 2 shows the inclusion flow chart.

The mean age ± standard deviation of the finally included patients with NLE was 29 ± 7 years (F: M 6:8), and 30 ± 7 years (F: M 12:15) in the control group, respectively. For NLE, the age of onset was 15 ± 9 years and the disease duration was 14 ± 8 years.

### Clinical data

Electroencephalography (EEG) detected seizure onset in the following locations: 1 left frontal, 5 left temporal, 1 left insular, 4 right frontal, 3 right temporal.

### Basic results

**Fig. 3:**
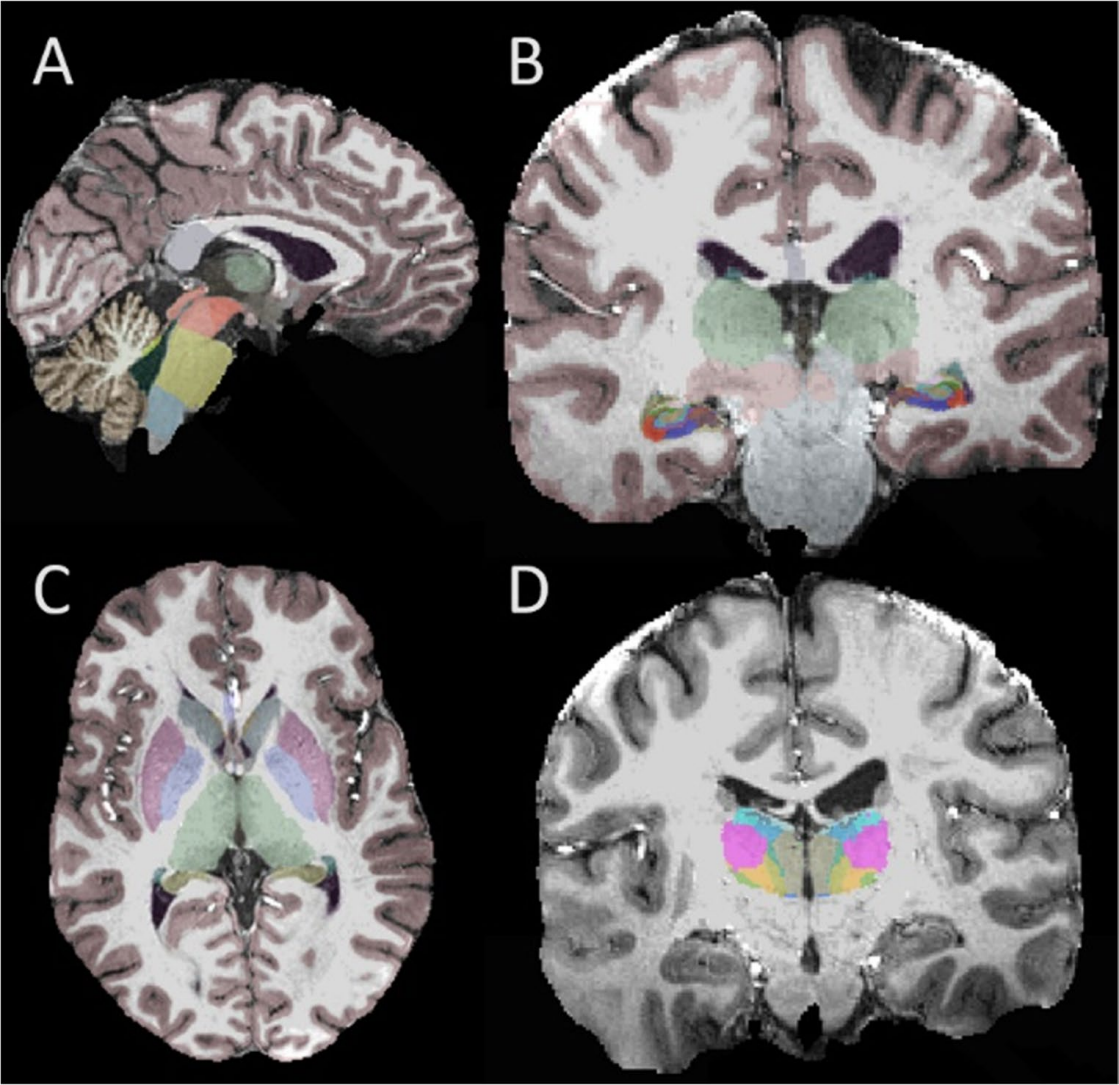
Segmentation examples in a healthy control individual (**A** – brainstem, **B** – hippocampal subfields and thalamic region, **C** – basal ganglia, **D** – thalamic nuclei)

We found a normal distribution in 89.3% of volumes of the control group and in 88.8% of the NLE group. Therefore, we used the non-parametric Mann-Whitney-U test for further analysis. Figure 3 demonstrates an example segmentation.

The comparison of total brain volumes revealed no significant differences (Left Focus vs. Control: *p* = 0.11; Right Focus vs. Control: *p* = 0.53).

Table 1 summarizes the mean volumes and standard deviations of the FastSurfer volumetry using the DKT atlas. For further analysis, volumes were normalized by dividing them by the whole brain volume. Interestingly, the standard deviations in patients with NLE were significantly higher than in the control group. The control group showed a slightly lower total brain volume.

**Table 1:** Mean volume ± standard deviation (mm^3^) of automated segmentation (ASEG) and DKT-Atlas

	Non-lesional epilepsy		Control group	
Brain region	Mean	Standard deviation	Mean	Standard deviation
BrainSeg	1,091,423	133,359	1,067,518	69,873
BrainSegNotVent	1,068,527	132,325	1,047,216	68,622
SupraTentorial	966,104	122,289	951,575	65,340
SupraTentorialNotVent	943,207	121,281	931,272	64,231
SubCortGray	57,535	5383	56,536	3380
Left-Cerebral-White-Matter	221,797	33,369	224,110	18,193
Left-Lateral-Ventricle	8591	3784	7877	3501
Left-Inf-Lat-Vent	460	210	440	142
Left-Cerebellum-White-Matter	12,477	1331	11,109	1608
Left-Cerebellum-Cortex	50,806	6095	47,331	4396
Left-Thalamus	7614	747	7637	579
Left-Caudate	3627	351	3665	339
Left-Putamen	5136	588	5050	381
Left-Pallidum	1955	190	2094	138
Left-Hippocampus	4073	498	3801	336
Left-Amygdala	1560	215	1357	107
Left-Accumbens-area	586	80	568	58
Left-VentralDC	4113	404	4113	312
Left-choroid-plexus	602	139	545	141
Right-Cerebral-White-Matter	223,068	33,783	222,629	18,399
Right-Lateral-Ventricle	7991	3764	7019	3395
Right-Inf-Lat-Vent	493	221	454	190
Right-Cerebellum-White-Matter	12,427	1363	11,458	1146
Right-Cerebellum-Cortex	49,610	5733	46,046	4214
Right-Thalamus	7389	758	7472	518
Right-Caudate	3773	580	3939	325
Right-Putamen	5163	642	5000	393
Right-Pallidum	1868	226	1758	148
Right-Hippocampus	4121	451	3796	324
Right-Amygdala	1721	244	1364	131
Right-Accumbens-area	621	132	574	68
Right-VentralDC	4217	440	4347	341
Right-choroid-plexus	731	168	674	141
WM-hypointensities	1412	416	1287	368
3rd-Ventricle	1086	473	859	333
4th-Ventricle	1751	584	1418	375
Brain-Stem	17,597	1951	16,654	1451
CSF	1191	288	1017	181
ctx-lh-caudalanteriorcingulate	2905	495	3077	443
ctx-lh-caudalmiddlefrontal	6423	1128	6871	991
ctx-lh-cuneus	4292	723	4393	732
ctx-lh-entorhinal	1497	214	971	213
ctx-lh-fusiform	7101	1234	5480	763
ctx-lh-inferiorparietal	11,017	2392	10,800	1132
ctx-lh-inferiortemporal	10,174	1637	8076	1218
ctx-lh-isthmuscingulate	2385	318	2315	354
ctx-lh-lateraloccipital	9695	1370	10,113	1107
ctx-lh-lateralorbitofrontal	8910	1074	7854	767
ctx-lh-lingual	6532	1121	5962	819
ctx-lh-medialorbitofrontal	4036	659	3117	420
ctx-lh-middletemporal	12,799	2187	12,218	1399
ctx-lh-parahippocampal	1761	230	1359	253
ctx-lh-paracentral	3724	616	4179	571
ctx-lh-parsopercularis	4055	705	4201	660
ctx-lh-parsorbitalis	2209	360	2278	339
ctx-lh-parstriangularis	4370	1005	4704	730
ctx-lh-pericalcarine	1914	334	2064	365
ctx-lh-postcentral	9479	1191	9640	1064
ctx-lh-posteriorcingulate	3105	575	3233	369
ctx-lh-precentral	11,739	1232	12,351	1138
ctx-lh-precuneus	8969	1544	8910	965
ctx-lh-rostralanteriorcingulate	3348	899	2950	480
ctx-lh-rostralmiddlefrontal	11,262	2077	12,323	1995
ctx-lh-superiorfrontal	23,337	2660	24,630	2803
ctx-lh-superiorparietal	9398	1455	9634	1136
ctx-lh-superiortemporal	16,286	1941	14,493	1306
ctx-lh-supramarginal	9083	1266	9139	1100
ctx-lh-transversetemporal	1027	178	1006	171
ctx-lh-insula	5745	798	5470	582
ctx-rh-caudalanteriorcingulate	1944	447	1956	392
ctx-rh-caudalmiddlefrontal	6057	1242	6626	1106
ctx-rh-cuneus	3945	512	3944	540
ctx-rh-entorhinal	1530	317	922	251
ctx-rh-fusiform	7131	1048	6070	794
ctx-rh-inferiorparietal	12,570	2205	12,995	1659
ctx-rh-inferiortemporal	10,244	1332	9003	1073
ctx-rh-isthmuscingulate	2116	308	2140	336
ctx-rh-lateraloccipital	10,887	1399	10,318	1032
ctx-rh-lateralorbitofrontal	8486	1262	6234	745
ctx-rh-lingual	6981	917	6340	793
ctx-rh-medialorbitofrontal	3989	599	2837	408
ctx-rh-middletemporal	12,823	2185	12,409	1045
ctx-rh-parahippocampal	1770	273	1309	199
ctx-rh-paracentral	3754	779	4250	485
ctx-rh-parsopercularis	4193	696	4249	513
ctx-rh-parsorbitalis	2508	325	2124	252
ctx-rh-parstriangularis	4049	755	4345	803
ctx-rh-pericalcarine	2104	345	2410	480
ctx-rh-postcentral	9159	1380	8793	1107
ctx-rh-posteriorcingulate	3263	503	3211	538
ctx-rh-precentral	11,265	1579	11,431	1248
ctx-rh-precuneus	9835	1823	9894	982
ctx-rh-rostralanteriorcingulate	2112	492	1653	396
ctx-rh-rostralmiddlefrontal	11,511	1994	12,587	1640
ctx-rh-superiorfrontal	26,471	3815	26,885	2999
ctx-rh-superiorparietal	9689	2142	9905	1044
ctx-rh-superiortemporal	15,281	1902	13,349	1256
ctx-rh-supramarginal	8522	1305	8549	951
ctx-rh-transversetemporal	835	156	765	125
ctx-rh-insula	5793	821	5399	576
Medulla	2707	281	2657	289
Pons	13,016	1681	12,224	1307
SCP	260	58	285	53
Midbrain	5760	497	5319	611
Whole_brainstem	21,744	2262	20,485	2095

Legend: lh – left hemisphere; rh – right hemisphere; ctx – cortex; DC – Diencephalon; SCP – superior cerebellar peduncle

Table 2 reveals the key results of the Mann-Whitney-U tests with significant volume alterations after Bonferroni correction.

**Table 2:** Significant values at a significance level of 5% (Bonferroni corrected p)

Structure (ipsilateral)	Left - *p* adjusted	Right - *p* adjusted
amygdala	1	**0.00096**
(whole_amygdala subscript)	1	1
ctx-entorhinal	**0.010**	**0.0035**
ctx-fusiform	**0.026**	0.59
ctx-lateralorbitofrontal	0.630	**0.00096**
ctx-medialorbitofrontal	**0.005**	**0.001504568**
ctx-parahippocampal	1	**0.010109272**
ctx-superiortemporal	1	**0.003496532**
hippocampal_tail	1	**0.010109272**
subiculum-body	1	**0.042145759**
hippocampal-fissure	1	**0.005079173**
presubiculum-body	**0.0336**	**0.001504568**
CL	**0.0015**	**0.042145759**

Legend: ctx – cortex; CL – central lateral nucleus of thalamus

As a major finding, we detected a significant volume loss of the central lateral nucleus of thalamus.

Additionally, we detected focal volume alterations on the focus side, both in the brain parenchyma and within the substructures. We noticed an enlargement of the ipsilateral presubiculum body. Significant volume increase was detected in the ipsilateral and contralateral entorhinal and medial orbitofrontal cortices. For right-sided epilepsy, we also found a volume alteration in the ipsilateral parahippocampal, inferiortemporal and superiortemporal cortices as well as in the ipsilateral fusiform gyrus for left-sided NLE.

Interestingly, an enlargement of the ipsi- and contralateral amygdala was detected by whole-brain volumetry, but this finding could not be confirmed in the amygdala subfield analysis.

The absolute values of key structures are listed in Table 3.

**Table 3:** Absolute values of the key structures

Structure	NLE group		Control group	
	Mean volume (mm)	SD (mm)	Mean volume (mm)	SD (mm)
whole_hippocampal_body (left)	1120	155	1042	78
whole_hippocampal_head (left)	1653	251	1527	147
ctx-entorhinal (left)	1497	214	967	202
ctx-medialorbitofrontal (left)	4036	659	3113	406
ctx-lateralorbitofrontal (left)	8910	1074	7762	822
ctx-fusiform (left)	7101	1234	5395	806
ctx-parahippocampal (left)	1761	230	1336	257
ctx-superiortemporal (left)	16,286	1941	14,414	1485
hippocampal_tail (left)	509	74	455	49
subiculum-body (left)	247	27	230	24
hippocampal-fissure (left)	207	33	180	19
presubiculum-body (left)	162	33	132	16
CA1-body (left)	154	28	134	23
CA3-body (left)	78	18	65	15
CA4-body (left)	120	18	111	11
GC-ML-DG-body (left)	133	18	123	12
CL (left)	32	5	39	6
whole_hippocampal_body (right)	1087	135	981	87
whole_hippocampal_head (right)	1681	228	1587	158
ctx-entorhinal (right)	1530	317	924	247
ctx-medialorbitofrontal (right)	3989	599	2845	414
ctx-lateralorbitofrontal (right)	8486	1262	6295	789
ctx-fusiform (right)	7131	1048	5956	815
ctx-parahippocampal (right)	1770	273	1300	190
ctx-superiortemporal (right)	15,281	1902	13,317	1297
hippocampal_tail (right)	499	65	386	56
subiculum-body (right)	228	23	195	21
hippocampal-fissure (right)	197	25	160	15
presubiculum-body (right)	123	19	98	13
CA1-body (right)	150	27	135	21
CA3-body (right)	90	17	68	13
CA4-body (right)	122	15	103	11
GC-ML-DG-body (right)	133	17	112	12
CL (right)	34	6	43	7

Legend: ctx – cortex; CL – central lateral nucleus of thalamus; CA – cornu ammonis; GC-ML-DG– Granular cell-molecular layer-dentate gyrus

The GLM analysis produced results like those obtained using the Mann-Whitney U test for most regions, except for the ipsilateral Central Lateral Nucleus (CL). Specifically, the Bonferroni-adjusted p-values for the ipsilateral CL ranged from 0.089 in patients with a left-sided focus to 0.098 in those with a right-sided focus. Detailed outcomes of the GLM analysis are provided in Supplementary Table 1.

The GLM analysis without skull-stripping segmented 12 patients (7 left, 5 right) and 17 controls, reducing power for left-sided foci. Consequently, few regions were significant after Bonferroni or FDR correction. For right-sided foci, significant regions largely matched the main analysis with skull-stripping. Although formal significance was not reached on the left, the affected regions corresponded to those in the main analysis, indicating that skull-stripping does not substantially alter the observed patterns. An overview can be found in Supplementary Table 2. Volume comparison showed greater variability in individuals without external skull-stripping. The mean Coefficient of Variation (CV) ± standard deviation for the epilepsy group was 19 ± 9% (median 17%) without external skull-stripping and 16 ± 7% (median 14%) with external skull-stripping, showing statistically significant different volumes in 10 (of 100) regions. In healthy controls, the CV was 20 ± 13% (median 16%) without external skull-stripping and 14 ± 7% (median 12%) with external skull-stripping, with significant differences observed in 41 of 100 tested regions. Detailed results are listed in Supplementary Table 3. The regions exhibiting significant differences were, as expected, those most strongly affected by artifacts, specifically the frontobasal, temporobasal, and brainstem areas.

### Sensitivity and Specificity/Area-under-Curves (AUC)

To test the ability to differentiate between the Healthy Control group and the non-lesional-epilepsy (NLE) group, we calculated the sensitivity, specificity, thresholds, AUC, and false positive rate for the detected key structures, which are summarized in Table 4.

**Table 4:** Receiver operating characteristics of key structures

Group	Structure	Area under curve	Sensitivity	Specificity	False-Positive-Rate
**Left-sided NLE**	presubiculum-body left	0.93	0.86	0.96	0.04
	CL left	0.97	1.00	0.96	0.04
	entorhinal cortex left	0.95	1.00	0.81	0.19
	medialorbitofrontal cortex left	0.96	0.86	0.96	0.04
	entorhinal cortex right	0.93	0.86	0.93	0.07
	medialorbitofrontal cortex right	0.98	1.00	0.93	0.07
**Right-sided NLE**	presubiculum-body right	0.97	1.00	0.89	0.11
	CL right	0.92	0.71	1.00	0.00
	entorhinal cortex right	0.96	1.00	0.93	0.07
	medialorbitofrontal cortex right	0.97	0.86	1.00	0.000
	entorhinal cortex left	0.97	0.86	0.96	0.04
	medialorbitofrontal cortex left	0.93	1.00	0.81	0.19
	medialorbitofrontal cortex right	0.98	1.00	0.923	0.07

Legend: NLE – non-lesional epilepsie; CL – central lateral nucleus of thalamus

**Fig. 4:**
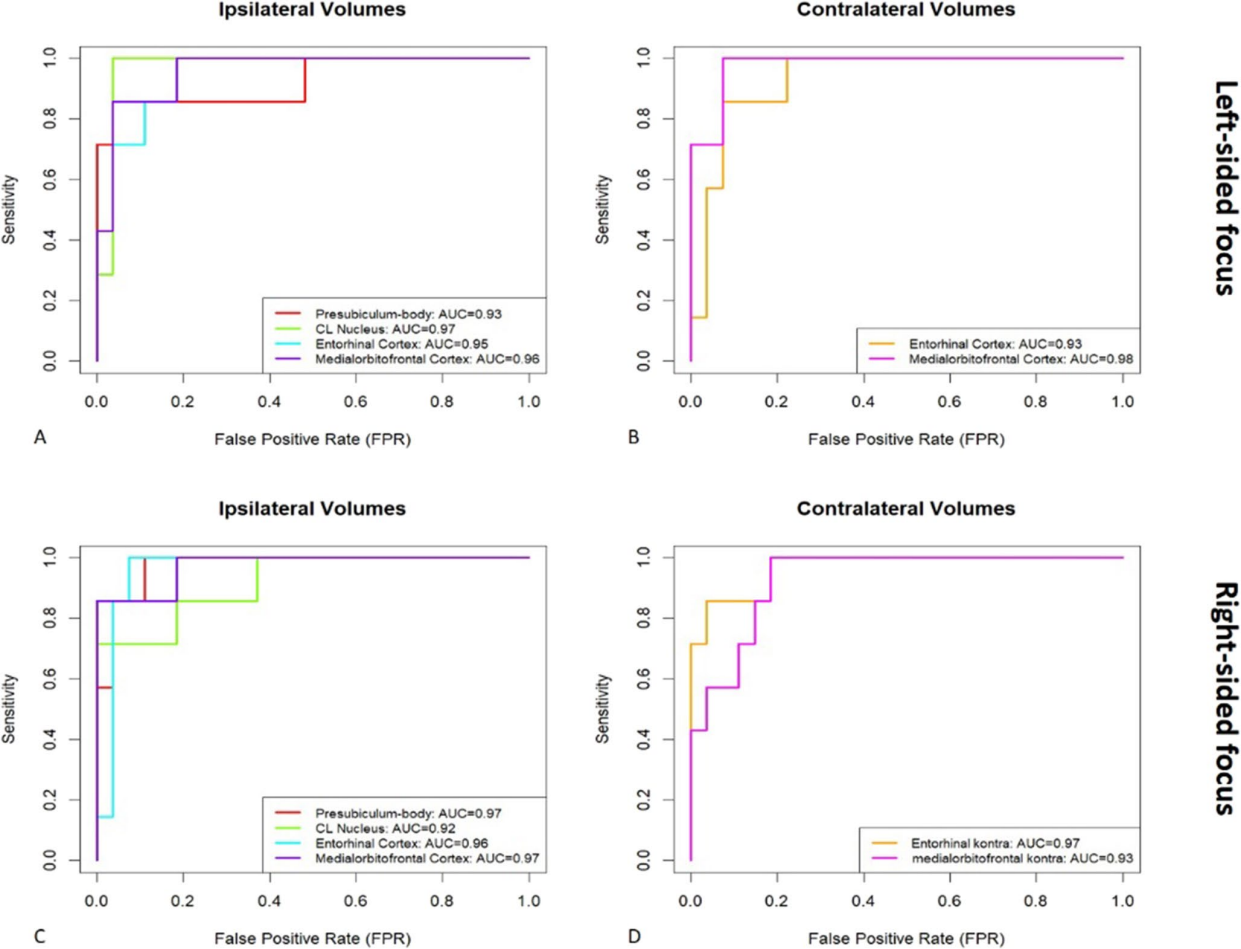
Receiver operation characteristics curves and area-under-curves of left-sided (**A**-**B**) and right-sided (**C**-**D**) non-lesional epilepsy for the ipsilateral presubiculum body, the central lateral nucleus of thalamus, as well as ipsilateral and contralateral entorhinal cortex and medialorbitofrontal cortex

Figure 4 shows the Receiver Operation Characteristics (ROC)-curves and the AUC.

**Fig. 5:**
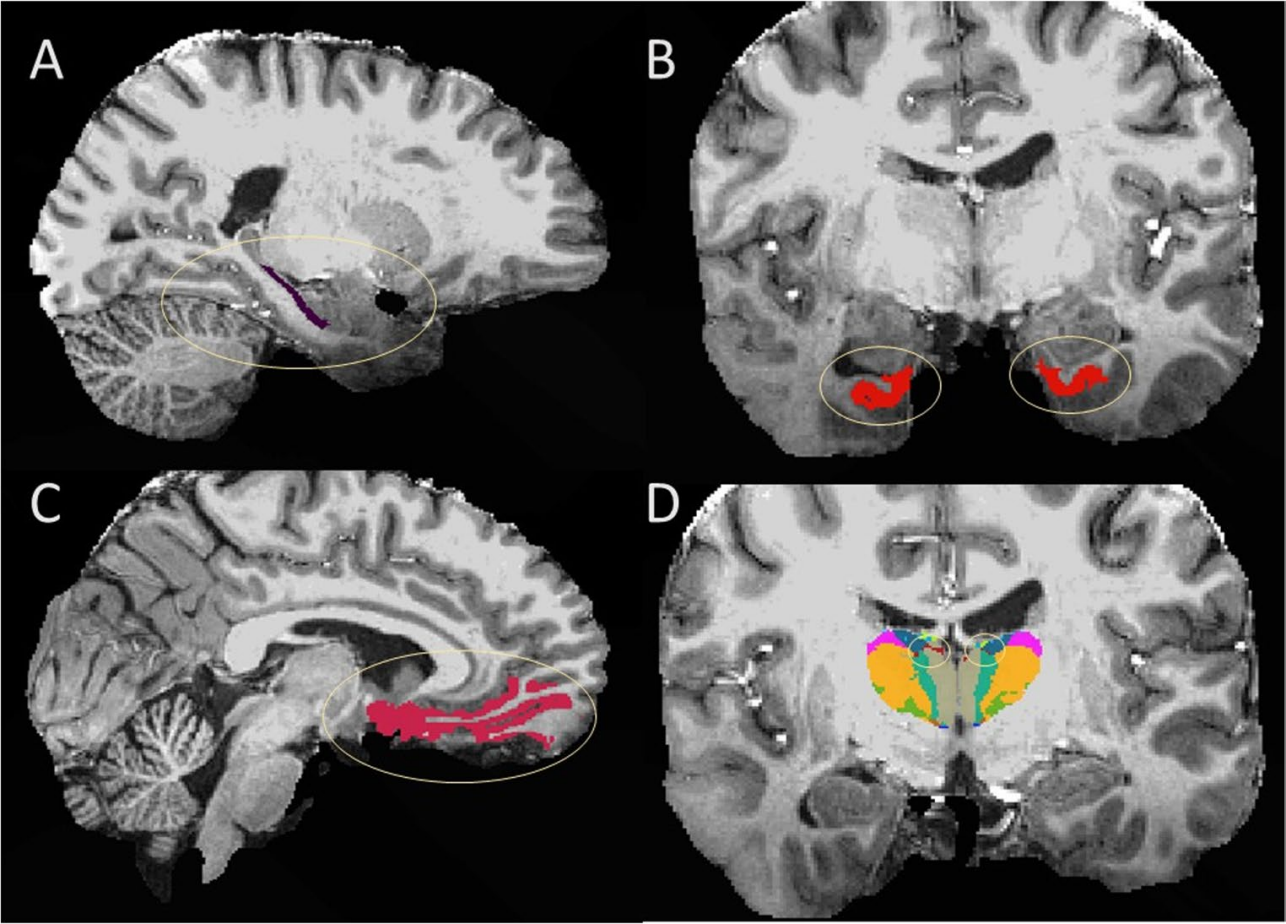
Example segmentation of key brain structures in a representative patient with non-lesional epilepsy (NLE): **A** – body of presubiculum; **B** – entorhinal cortex; **C** – medialorbitofrontal cortex; **D** – central lateral nucleus

The evaluated key structures are marked in Fig. 5.

To evaluate the ability of structural features to differentiate left- from right-sided focus, we performed an additional ROC analysis. Fifteen structures achieved an AUC above 0.9, with the five best-performing structures shown in Supplementary Fig. 1.

### Confounders

Even though the T1 MPRAGE sequence parameters were identical, an independent confounding factor is the date of the MRI. Since the control group was scanned as part of an earlier study, the MRIs were on average 3 years older.

## Discussion

In this first-time 7 T volumetric analysis of the selected patient cohort, which included 14 patients with NLE and 27 controls, we identified focal volume alterations in the seizure onset hemisphere. These alterations affected both the brain parenchyma and the hippocampal subfields, extending from the central nucleus and presubiculum to the amygdala and various cortical regions. Remarkably, we observed a volume decrease in the CL nucleus, which, to the best of our knowledge, has not been reported previously.

### Thalamus

Thalamus volumetry revealed significant volume loss in the ipsilateral CL for both, left-sided and right-sided seizure onset zones. This is most interesting, as CL activation has been linked to a shift in the level of consciousness and control transitions from sleep to wakefulness (Redingbaugh 2020). Larkum (1999) and Jones (2009) reasoned that connectivity between layer V pyramidal neurons and the intralaminar nuclei, including CL, is the responsible mechanism for synchronization within the cortical column (Llinas et al. 1998, Shiff 2020 for a review). The finding of seizure laterality dependent atrophy in CL might therefore hint towards a pathophysiological mechanism related to clinical features of epilepsy such as postictal drowsiness or confusion. Interestingly, the centromedian (CM) nucleus, which is also part of the intralaminar nuclei and exhibits connectivity patterns similar to those of the CL nucleus [17], has been extensively studied and serves as a target for therapeutic interventions, including deep brain stimulation [18, 19].

Although the GLM analysis did not confirm the findings of the Mann-Whitney U test, a decrease in the volume of the Centro lateral Nucleus (CL) remains plausible. However, given our small patient cohort, the influence of covariates and the application of the Bonferroni correction in the GLM may have affected the statistical significance of these results.

### Hippocampal subfields

Hippocampal subfield volumetry revealed an ipsilateral volume increase in the presubiculum body. In right-sided NLE, the ipsilateral hippocampal tail and fissure as well as the subiculum also showed a significant volume increase. Volume changes in the hippocampus are a known finding in a wide range of neurological disorders. In epilepsy with hippocampal sclerosis (HS), early patterns could be detected by 7 T MRI [20]. Furthermore volumetry exhibits neuronal loss in the cornu ammonis (CA1) and CA4 in HS Type 1, whereas HS Type 2 shows neuronal loss predominantly in CA1, and HS Type 3 is characterized by volume loss in CA4 [21]. Other studies have revealed alterations in volume and microstructures of dentate gyrus, CA1, CA3, and the subiculum in patients with HS on 3 T [22, 23]. Even a correlation between volumetry and histopathological cell count has been confirmed [24]. Higher field-strengths could improve the hippocampus imaging, and therefore enable a better radiological grading of HS [25]. A study involving four patients with HS has already demonstrated the feasibility of 7 T MRI and its capability to provide higher spatial resolution and improved tissue contrast [26]. In addition, a volumetric study of 2911 patients with neurodegenerative disorders [27] reveals an morphological change in the hippocampal region, e.g. CA1 is one of the first targets of Alzheimer’s disease [28]. In a study from Brazil, CA1 was shown to be atrophied in patients with temporal lobe epilepsy (TLE) [29]. Nevertheless, in some patients, normal volume in CA1 can be maintained despite neuronal volume loss [30]. In children with drug-resistant epilepsy, neither total brain volume nor hippocampal volume appear to change during follow-up [31]. Moreover, seizure control does not seem to predict changes in hippocampal volume [31]. A FreeSurfer 3 T study in temporal lobe epilepsy could not detect any significant volume loss of the hippocampal subfields in a subgroup of patients with NLE [32]. Relevant to our study, a US study revealed a volume loss in the body of the presubiculum in patients with ongoing seizures, which aligns with our findings [33]. Although most studies report hippocampal atrophy, there is evidence suggesting that, in association with interictal epileptiform discharges (IEDs), hippocampal structures can be enlarged on the ipsilateral side [34].

With improved T1 sequences, such as MP2RAGE, 7 T MRI could not only establish itself in the search for focal cortical dysplasias or microgyria [35] but also enable enhanced volumetric analysis of smaller structures, such as the hippocampal subfields.

### Additional regions of interest

Interestingly, an enlargement of the ipsi-and contralateral amygdala was detected by the whole brain volumetry, but could not be confirmed in the amygdala subfield analysis. Besides the amygdala, we found an enlargement of the ipsilateral and contralateral entorhinal and medial orbitofrontal cortices.

Ipsilateral atrophy of the amygdala has been well-documented in patients with temporal lobe epilepsy (TLE) accompanied by hippocampal sclerosis (HS), suggesting an underlying process of neuronal loss [36, 37]. Intriguingly, Bernhardt et al. (2014) revealed that certain subgroups of TLE may exhibit bilateral hypertrophy of the amygdala, challenging the conventional understanding of this structure’s role in epilepsy. Remarkably, some patients with imaging-negative TLE have been found to display isolated ipsilateral amygdala enlargement [38]. In addition to the amygdala, the entorhinal cortex has garnered attention in several studies. These investigations have identified ipsilateral atrophy [39] and have observed a significant connection between the hippocampal gyrus and the subiculum with the entorhinal cortex in TLE patients [40, 41]. In contrast, the medial orbitofrontal cortex has been relatively underexplored. However, Feng et al. has demonstrated increased connectivity of the orbitofrontal cortex with other epilepsy-relevant structures in TLE patients [42], while Coan et al. reported subtle atrophy in patients with TLE without HS [43].

Furthermore, our findings reveal an increase in the ipsilateral parahippocampal, inferior temporal, and superior temporal cortices in patients with right-sided epilepsy, as well as in the ipsilateral fusiform gyrus in those with left-sided epilepsy. This could be attributed to the fact that, in the early phases of neuronal degeneration, an edematous remodeling of these structures leads to a subtle increase in volume.

Overall, our results align with existing literature but also present discrepancies in certain regions, potentially reflecting the diverse spectrum of epileptic foci present in our patient cohort. An enlargement of the amygdala has already been described, which could be due to a secondary reactive process to seizures in the epileptogenic temporal lobe [44]. The presubiculum plays a critical role in spatial navigation, spatial representation [45] and head-direction [46].

Although it remains unclear whether these volume alterations are a consequence or a cause of epilepsy, volumetric analysis serves as a valuable tool for screening and comparison with EEG to confirm the focus side.

### Limitations

Major limitation of our study is the small patient’s number. The visual detection of epileptogenic lesions, which may be better differentiated due to the higher resolution in 7 T, was not part of this study.

A potential confounder was that the 7 T MRI scans for the control group were conducted, on average, three years earlier than those for the epilepsy group. This means that although the sequence parameters remained unchanged, technical nuances may have been influenced by routine maintenance procedures such as system updates. However, no major changes to the coil or scanner hardware occurred. This could potentially explain the volumetric differences in the cortices, which may be affected by susceptibility artifacts, especially the entorhinal and medial orbitofrontal cortices. The same effect may explain the higher impact of skull-stripping on the results of the control group. Our positive test for normal distribution, however, showed that there were no significant deviations within the control group.

While large studies [47, 48] subdivided their cohorts into temporal and extratemporal groups and included specific patient parameters such as disease duration as well as pathology-specific factors in their analyses, we did not perform such a separation, primarily due to our limited sample size.

In our study, regional volumes were normalized to Total Brain Volume (TBV). Although Total Intracranial Volume (TIV) and regression-based covariate approaches have been shown to perform better in certain contexts (Opfer et al., 2022; Wang et al., 2024, particularly in studies of diseases accompanied by global brain atrophy, we considered TBV more appropriate for our cohort, where such atrophy is unlikely. In addition, TIV has been shown to be slightly influenced by TBV itself [49], which further supports our choice.

Another limitation is the use of the MPRAGE sequence, while the novel MP2RAGE sequence offers several advantages, making it a potential candidate to replace MPRAGE in the future [35].

### Outlook

The image quality in the 7 T is improving slowly, but steadily, while the image quality in the 3 T already seems to have reached its peak. New techniques as parallel transmission [50] and TR-FOCI impulses [51] are currently improving the 7 T imaging.

MPR2RAGE is a more time-intensive yet higher-contrast alternative [52]. Another approach are more myelin sensitive sequences or the further development of post-processing procedures [53].

With advances in imaging technology enhancing image quality and segmentation accuracy, volumetric measurements of specific target structures may become reliable diagnostic criteria for patients with epileptic seizures, even when conventional evidence is lacking. Our findings of high sensitivity and specificity are encouraging but require validation in larger-scale studies.

## Conclusion

Significant volumetric alterations were identified in six brain regions when comparing a cohort of patients with non-lesional epilepsy to healthy controls, largely corroborating findings reported in other epilepsy subtypes. These regions exhibited favorable sensitivity and specificity, indicating the potential utility of volumetry as a diagnostic criterion. Of particular interest, volume loss was observed in the ipsilateral central lateral nucleus of the thalamus—a key component of the intralaminar nuclei involved in regulating consciousness and cortical excitability—alongside volume increases in the ipsilateral presubiculum-body, both of which are infrequently documented in the literature. To enhance the robustness and generalizability of these findings, further studies with larger patient cohorts are warranted.

## Supplementary Information

Below is the link to the electronic supplementary material.

Supplementary Material 1

Supplementary Material 2

Supplementary Material 3

Supplementary Material 4

## Data Availability

The datasets used and/or analyzed during the current study are available from the corresponding author on reasonable request.

## Abbreviations

AUC: area under curves
CA: cornu ammonis
CL: central lateral nucleus (of the thalamus)
CTX: cortex
DC: diencephalon
GC-ML-DG: granular cell-molecular layer-dentate gyrus
GLM: general linear model
HS: hippocampal sclerosis
MRI: magnetic resonance imaging
MR: magnetic resonance
NLE: non-lesional epilepsy
ROC: receiver operating characteristics
SCP: superior cerebellar peduncle
SD: standard deviation
