# Supplementary material for: Local brain volume reductions in patients with non-lesional epilepsy on 7T MRI
Source: Neuroradiology. 2025 Nov 18;68(6):1439–52. doi: 10.1007/s00234-025-03843-3 (PMC13323624; doi:10.1007/s00234-025-03843-3)

**Supplementary Figure 1**

ROC analysis of the top five brain structures demonstrating the highest diagnostic capability for identifying the epileptogenic (focus) hemisphere.
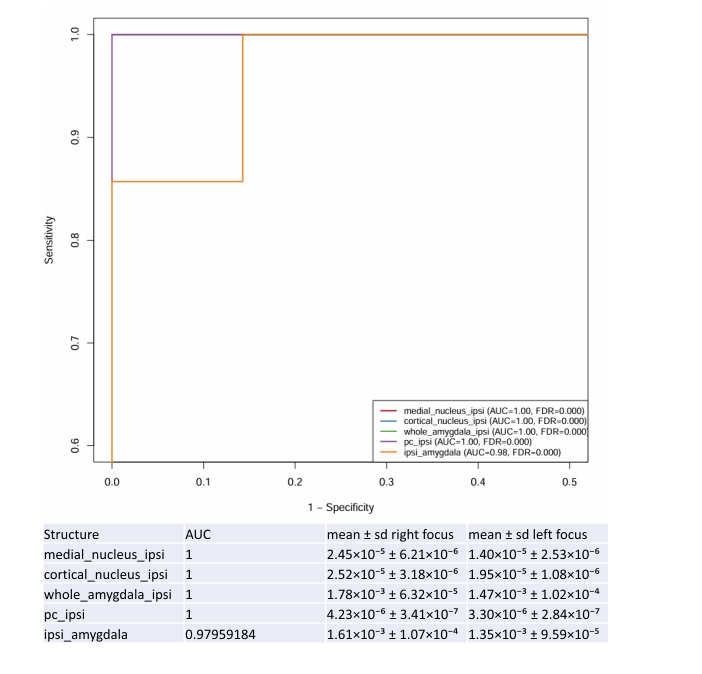

Supplement: Supplementary file 1 — Supplementary Material 1 [file 234_2025_3843_MOESM1_ESM.docx]
