# Supplementary material for: Local brain volume reductions in patients with non-lesional epilepsy on 7T MRI
Source: Neuroradiology. 2025 Nov 18;68(6):1439–52. doi: 10.1007/s00234-025-03843-3 (PMC13323624; doi:10.1007/s00234-025-03843-3)
Supplement: Supplementary file 2 — Supplementary Material 2 [file 234_2025_3843_MOESM2_ESM.docx]

**Supplementary Table 1**

GLM analysis of epilepsy with a left-sided focus compared to healthy controls, using age and gender as covariates

| **Region** | **Method** | **Beta** | **SE** | **t_value** | **p_value** | **resid_sw_p** | **adj_p_bonferroni** |
| --- | --- | --- | --- | --- | --- | --- | --- |
| Left-Cerebral-White-Matter () | Gaussian | -0.006832761 | 0.003533544 | -1.93369 | 0.062635973 | 0.161694 | 1 |
| Left-Lateral-Ventricle () | Gaussian | 0.002132538 | 0.001531185 | 1.392737 | 0.173935961 | 0.00012 | 1 |
| Left-Inf-Lat-Vent () | Gaussian | 7.10234E-05 | 6.47746E-05 | 1.096471 | 0.281596852 | 0.231291 | 1 |
| Left-Cerebellum-White-Matter () | Gaussian | 0.000721803 | 0.000663012 | 1.088673 | 0.284966994 | 0.636955 | 1 |
| Left-Cerebellum-Cortex () | Gaussian | 0.002042991 | 0.001395081 | 1.464424 | 0.15347945 | 0.686398 | 1 |
| Left-Thalamus () | Gaussian | -0.000168634 | 0.00013297 | -1.26821 | 0.214477529 | 0.871091 | 1 |
| Left-Caudate () | Gaussian | -0.000246942 | 0.000123081 | -2.00635 | 0.053907729 | 0.798942 | 1 |
| Left-Putamen () | Gaussian | -0.000183256 | 0.000146841 | -1.24799 | 0.221687652 | 0.645024 | 1 |
| Left-Pallidum () | Gaussian | -0.000178817 | 5.4015E-05 | -3.31051 | 0.002431764 | 0.715827 | 0.517966 |
| Left-Hippocampus () | Gaussian | 0.000151831 | 0.000119463 | 1.270947 | 0.213514654 | 0.153397 | 1 |
| Left-Amygdala () | Gaussian | 8.34437E-05 | 4.93076E-05 | 1.69231 | 0.100949522 | 0.650847 | 1 |
| Left-Accumbens-area () | Gaussian | -1.12896E-05 | 2.36978E-05 | -0.4764 | 0.637243123 | 0.551054 | 1 |
| Left-VentralDC () | Gaussian | -0.000111421 | 0.000101541 | -1.0973 | 0.2812393 | 0.20083 | 1 |
| Left-choroid-plexus () | Gaussian | 5.77397E-05 | 5.27033E-05 | 1.095562 | 0.281987936 | 0.958182 | 1 |
| Right-Cerebral-White-Matter () | Gaussian | -0.003895098 | 0.003505965 | -1.11099 | 0.275397182 | 0.275726 | 1 |
| Right-Lateral-Ventricle () | Gaussian | 0.002092093 | 0.001474864 | 1.418499 | 0.166350858 | 6.55E-06 | 1 |
| Right-Inf-Lat-Vent () | Gaussian | 6.99807E-05 | 8.70897E-05 | 0.803547 | 0.427980132 | 0.000205 | 1 |
| Right-Cerebellum-White-Matter () | Gaussian | 0.000340174 | 0.000484514 | 0.702093 | 0.488033133 | 0.710311 | 1 |
| Right-Cerebellum-Cortex () | Gaussian | 0.001929004 | 0.001475187 | 1.307634 | 0.200930399 | 0.944187 | 1 |
| Right-Thalamus () | Gaussian | -0.000150245 | 0.000150896 | -0.99569 | 0.327365787 | 0.395051 | 1 |
| Right-Caudate () | Gaussian | -0.000260588 | 0.000128535 | -2.02736 | 0.051590215 | 0.692374 | 1 |
| Right-Putamen () | Gaussian | -6.12985E-05 | 0.000133021 | -0.46082 | 0.648253202 | 0.896353 | 1 |
| Right-Pallidum () | Gaussian | 6.95014E-05 | 5.55987E-05 | 1.250055 | 0.2209431 | 0.657571 | 1 |
| Right-Hippocampus () | Gaussian | 0.00017683 | 0.000114746 | 1.541063 | 0.133784691 | 0.561004 | 1 |
| Right-Amygdala () | Gaussian | 0.000260547 | 6.38559E-05 | 4.080238 | 0.000306141 | 0.738522 | 0.065208 |
| Right-Accumbens-area () | Gaussian | 2.63435E-05 | 3.03946E-05 | 0.866718 | 0.39297579 | 0.200065 | 1 |
| Right-VentralDC () | Gaussian | -0.000220041 | 0.000111621 | -1.97132 | 0.057972214 | 0.241203 | 1 |
| Right-choroid-plexus () | Gaussian | 0.00010119 | 5.13859E-05 | 1.969221 | 0.058224422 | 0.473306 | 1 |
| Brain-Stem () | Gaussian | -0.000243789 | 0.000559131 | -0.43601 | 0.665948353 | 0.672118 | 1 |
| CSF () | Gaussian | 0.000190915 | 7.81738E-05 | 2.44218 | 0.020706746 | 0.110101 | 1 |
| ctx-lh-caudalanteriorcingulate () | Gaussian | -0.000128699 | 0.000130142 | -0.98891 | 0.330617674 | 0.868901 | 1 |
| ctx-lh-caudalmiddlefrontal () | Gaussian | -0.000523408 | 0.000358882 | -1.45844 | 0.155109875 | 0.302626 | 1 |
| ctx-lh-cuneus () | Gaussian | -0.000220598 | 0.000306007 | -0.72089 | 0.476556355 | 0.41035 | 1 |
| ctx-lh-entorhinal () | Gaussian | 0.000428769 | 9.24264E-05 | 4.639033 | 6.44366E-05 | 0.083506 | 0.013725 |
| ctx-lh-fusiform () | Gaussian | 0.001436511 | 0.00029622 | 4.849468 | 3.56271E-05 | 0.224032 | 0.007589 |
| ctx-lh-inferiorparietal () | Gaussian | -7.59867E-05 | 0.000485012 | -0.15667 | 0.87655476 | 0.631482 | 1 |
| ctx-lh-inferiortemporal () | Gaussian | 0.001267614 | 0.000393284 | 3.223148 | 0.003050811 | 0.648443 | 0.649823 |
| ctx-lh-isthmuscingulate () | Gaussian | 3.95303E-05 | 0.000106744 | 0.37033 | 0.713738835 | 0.433248 | 1 |
| ctx-lh-lateraloccipital () | Gaussian | -0.000752569 | 0.000436069 | -1.7258 | 0.094670914 | 0.327945 | 1 |
| ctx-lh-lateralorbitofrontal () | Gaussian | 0.000817653 | 0.000256518 | 3.187504 | 0.00334452 | 0.983015 | 0.712383 |
| ctx-lh-lingual () | Gaussian | 0.000474564 | 0.000315249 | 1.505362 | 0.142688322 | 0.952292 | 1 |
| ctx-lh-medialorbitofrontal () | Gaussian | 0.000841245 | 0.000153141 | 5.493279 | 5.78719E-06 | 0.293016 | 0.001233 |
| ctx-lh-middletemporal () | Gaussian | 0.000283509 | 0.000478032 | 0.593077 | 0.55757476 | 0.724222 | 1 |
| ctx-lh-parahippocampal () | Gaussian | 0.000343398 | 0.000120441 | 2.851168 | 0.007808067 | 0.305089 | 1 |
| ctx-lh-paracentral () | Gaussian | -0.00071085 | 0.000191337 | -3.71518 | 0.000829899 | 0.529935 | 0.176768 |
| ctx-lh-parsopercularis () | Gaussian | -0.000246601 | 0.000246628 | -0.99989 | 0.325360522 | 0.893269 | 1 |
| ctx-lh-parsorbitalis () | Gaussian | -5.69844E-06 | 0.00012039 | -0.04733 | 0.962561536 | 0.628247 | 1 |
| ctx-lh-parstriangularis () | Gaussian | -0.000587623 | 0.000310054 | -1.89523 | 0.067734241 | 0.807595 | 1 |
| ctx-lh-pericalcarine () | Gaussian | -0.000122537 | 0.000145785 | -0.84054 | 0.407257697 | 0.101039 | 1 |
| ctx-lh-postcentral () | Gaussian | 0.000120638 | 0.000358457 | 0.336548 | 0.738800522 | 0.314648 | 1 |
| ctx-lh-posteriorcingulate () | Gaussian | -0.000269822 | 0.000137307 | -1.9651 | 0.058721174 | 0.217561 | 1 |
| ctx-lh-precentral () | Gaussian | -0.001124323 | 0.000400286 | -2.8088 | 0.008664543 | 0.777798 | 1 |
| ctx-lh-precuneus () | Gaussian | -8.6092E-05 | 0.000351017 | -0.24526 | 0.807921922 | 0.80181 | 1 |
| ctx-lh-rostralanteriorcingulate () | Gaussian | 0.000641596 | 0.000176264 | 3.639972 | 0.001016212 | 0.259828 | 0.216453 |
| ctx-lh-rostralmiddlefrontal () | Gaussian | -0.001331735 | 0.000645257 | -2.06388 | 0.047771645 | 0.238533 | 1 |
| ctx-lh-superiorfrontal () | Gaussian | -0.001962548 | 0.000681329 | -2.88047 | 0.007263063 | 0.917215 | 1 |
| ctx-lh-superiorparietal () | Gaussian | -0.000482418 | 0.000389251 | -1.23935 | 0.224825043 | 0.677437 | 1 |
| ctx-lh-superiortemporal () | Gaussian | 0.000851718 | 0.000394745 | 2.157642 | 0.039087728 | 0.218191 | 1 |
| ctx-lh-supramarginal () | Gaussian | -0.00013535 | 0.000351234 | -0.38536 | 0.70269283 | 0.228035 | 1 |
| ctx-lh-transversetemporal () | Gaussian | -5.23648E-05 | 5.43162E-05 | -0.96407 | 0.342716856 | 0.196637 | 1 |
| ctx-lh-insula () | Gaussian | 5.17084E-05 | 0.000174356 | 0.296568 | 0.768839826 | 0.687404 | 1 |
| ctx-rh-caudalanteriorcingulate () | Gaussian | -1.41803E-05 | 0.000138902 | -0.10209 | 0.919365976 | 0.630725 | 1 |
| ctx-rh-caudalmiddlefrontal () | Gaussian | -0.000731936 | 0.000368113 | -1.98834 | 0.055964556 | 0.13015 | 1 |
| ctx-rh-cuneus () | Gaussian | 4.07281E-05 | 0.000217787 | 0.187009 | 0.85291265 | 0.609727 | 1 |
| ctx-rh-entorhinal () | Gaussian | 0.000491513 | 0.000113962 | 4.312969 | 0.000160545 | 0.136099 | 0.034196 |
| ctx-rh-fusiform () | Gaussian | 0.000934777 | 0.000288885 | 3.235808 | 0.002952564 | 0.750482 | 0.628896 |
| ctx-rh-inferiorparietal () | Gaussian | -0.000823241 | 0.000575862 | -1.42958 | 0.163169519 | 0.394082 | 1 |
| ctx-rh-inferiortemporal () | Gaussian | 0.000780881 | 0.000406479 | 1.921083 | 0.064268756 | 0.699795 | 1 |
| ctx-rh-isthmuscingulate () | Gaussian | -4.19427E-05 | 0.000119272 | -0.35166 | 0.727554598 | 0.226213 | 1 |
| ctx-rh-lateraloccipital () | Gaussian | 0.000126832 | 0.000334752 | 0.378883 | 0.707443262 | 0.325405 | 1 |
| ctx-rh-lateralorbitofrontal () | Gaussian | 0.002145933 | 0.000271336 | 7.90878 | 7.93958E-09 | 0.615515 | 1.69E-06 |
| ctx-rh-lingual () | Gaussian | 0.00038546 | 0.000293211 | 1.314616 | 0.198601156 | 0.257166 | 1 |
| ctx-rh-medialorbitofrontal () | Gaussian | 0.001077248 | 0.000170834 | 6.305813 | 5.95005E-07 | 0.370683 | 0.000127 |
| ctx-rh-middletemporal () | Gaussian | 0.000147136 | 0.000361222 | 0.407328 | 0.686659005 | 0.254455 | 1 |
| ctx-rh-parahippocampal () | Gaussian | 0.000393006 | 9.79385E-05 | 4.012784 | 0.000368658 | 0.843512 | 0.078524 |
| ctx-rh-paracentral () | Gaussian | -0.000564617 | 0.000208012 | -2.71435 | 0.010900906 | 0.527654 | 1 |
| ctx-rh-parsopercularis () | Gaussian | -0.000100007 | 0.000193573 | -0.51663 | 0.609199043 | 0.185028 | 1 |
| ctx-rh-parsorbitalis () | Gaussian | 0.000285479 | 0.000103169 | 2.767097 | 0.009592974 | 0.078364 | 1 |
| ctx-rh-parstriangularis () | Gaussian | -0.000458194 | 0.000281208 | -1.62938 | 0.113689807 | 0.068646 | 1 |
| ctx-rh-pericalcarine () | Gaussian | -0.000296251 | 0.000169596 | -1.7468 | 0.090904677 | 0.963394 | 1 |
| ctx-rh-postcentral () | Gaussian | 0.000413851 | 0.000448391 | 0.922969 | 0.363387477 | 0.306037 | 1 |
| ctx-rh-posteriorcingulate () | Gaussian | 8.79597E-05 | 0.000192854 | 0.456096 | 0.65160584 | 0.795311 | 1 |
| ctx-rh-precentral () | Gaussian | -0.000469966 | 0.000395236 | -1.18908 | 0.243736579 | 0.335546 | 1 |
| ctx-rh-precuneus () | Gaussian | -0.000583263 | 0.000330432 | -1.76515 | 0.087717071 | 0.228856 | 1 |
| ctx-rh-rostralanteriorcingulate () | Gaussian | 0.000558764 | 0.000151983 | 3.67648 | 0.000921183 | 0.456688 | 0.196212 |
| ctx-rh-rostralmiddlefrontal () | Gaussian | -0.001082175 | 0.000543334 | -1.99173 | 0.055572632 | 0.890773 | 1 |
| ctx-rh-superiorfrontal () | Gaussian | -0.001331299 | 0.000794389 | -1.67588 | 0.104155577 | 0.413905 | 1 |
| ctx-rh-superiorparietal () | Gaussian | -0.000152729 | 0.000413652 | -0.36922 | 0.714555846 | 0.536177 | 1 |
| ctx-rh-superiortemporal () | Gaussian | 0.000810359 | 0.000349916 | 2.315868 | 0.02758444 | 0.268478 | 1 |
| ctx-rh-supramarginal () | Gaussian | -0.000150198 | 0.0003166 | -0.47441 | 0.638642647 | 0.21904 | 1 |
| ctx-rh-transversetemporal () | Gaussian | 2.40277E-06 | 4.8108E-05 | 0.049945 | 0.960497045 | 0.56259 | 1 |
| ctx-rh-insula () | Gaussian | 0.000180645 | 0.000183531 | 0.984277 | 0.332851452 | 0.64766 | 1 |
| Medulla () | Gaussian | -6.55766E-05 | 0.000137688 | -0.47627 | 0.637331683 | 0.50967 | 1 |
| Pons () | Gaussian | -0.00026774 | 0.000551902 | -0.48512 | 0.631113425 | 0.677215 | 1 |
| SCP () | Gaussian | -2.12454E-05 | 2.03202E-05 | -1.04553 | 0.304128362 | 0.436509 | 1 |
| Midbrain () | Gaussian | 0.000231854 | 0.00026626 | 0.87078 | 0.390789414 | 0.40084 | 1 |
| Whole_brainstem () | Gaussian | -0.000122708 | 0.000900772 | -0.13622 | 0.89255352 | 0.469047 | 1 |
| Lateral-nucleus (lh) | Gaussian | 6.36906E-07 | 2.55386E-05 | 0.024939 | 0.980268845 | 0.774056 | 1 |
| Basal-nucleus (lh) | Gaussian | 1.62837E-05 | 1.46862E-05 | 1.108775 | 0.276337325 | 0.485608 | 1 |
| Accessory-Basal-nucleus (lh) | Gaussian | 1.414E-05 | 8.13244E-06 | 1.738715 | 0.092339446 | 0.577871 | 1 |
| Anterior-amygdaloid-area-AAA (lh) | Gaussian | -4.06136E-07 | 3.32065E-06 | -0.12231 | 0.903472171 | 0.952826 | 1 |
| Central-nucleus (lh) | Gaussian | 3.37285E-06 | 1.6473E-06 | 2.047498 | 0.049452876 | 0.924275 | 1 |
| Medial-nucleus (lh) | Gaussian | -1.12601E-07 | 1.29041E-06 | -0.08726 | 0.931045268 | 0.896853 | 1 |
| Cortical-nucleus (lh) | Gaussian | 4.56395E-07 | 9.77193E-07 | 0.467047 | 0.643840508 | 0.545359 | 1 |
| Corticoamygdaloid-transitio (lh) | Gaussian | 1.69176E-06 | 4.80771E-06 | 0.351885 | 0.727384459 | 0.434437 | 1 |
| Paralaminar-nucleus (lh) | Gaussian | 1.00686E-06 | 1.62976E-06 | 0.617799 | 0.54136999 | 0.323875 | 1 |
| Whole_amygdala (lh) | Gaussian | 3.70698E-05 | 5.71582E-05 | 0.648546 | 0.521565591 | 0.293893 | 1 |
| Lateral-nucleus (rh) | Gaussian | 1.23692E-06 | 3.85478E-05 | 0.032088 | 0.97461444 | 0.622627 | 1 |
| Basal-nucleus (rh) | Gaussian | 4.31166E-05 | 1.73932E-05 | 2.478938 | 0.019022903 | 0.773413 | 1 |
| Accessory-Basal-nucleus (rh) | Gaussian | 2.28516E-05 | 9.19336E-06 | 2.485665 | 0.018728702 | 0.924152 | 1 |
| Anterior-amygdaloid-area-AAA (rh) | Gaussian | 2.01089E-06 | 2.98153E-06 | 0.674449 | 0.505190366 | 0.393925 | 1 |
| Central-nucleus (rh) | Gaussian | 4.99503E-06 | 2.28027E-06 | 2.190544 | 0.036391803 | 0.660181 | 1 |
| Medial-nucleus (rh) | Gaussian | 4.89781E-07 | 1.60297E-06 | 0.305546 | 0.762060614 | 0.895072 | 1 |
| Cortical-nucleus (rh) | Gaussian | 2.44036E-06 | 1.12415E-06 | 2.170853 | 0.03798452 | 0.384419 | 1 |
| Corticoamygdaloid-transitio (rh) | Gaussian | 2.48018E-06 | 7.203E-06 | 0.344326 | 0.733002899 | 0.518071 | 1 |
| Paralaminar-nucleus (rh) | Gaussian | 3.3113E-06 | 1.94351E-06 | 1.703776 | 0.098761865 | 0.947025 | 1 |
| Whole_amygdala (rh) | Gaussian | 8.29327E-05 | 7.39862E-05 | 1.120921 | 0.271214643 | 0.664074 | 1 |
| Hippocampal_tail (lh) | Gaussian | 3.80597E-05 | 1.94835E-05 | 1.953435 | 0.060149537 | 0.790326 | 1 |
| subiculum-body (lh) | Gaussian | 1.59398E-05 | 6.98284E-06 | 2.282707 | 0.029704874 | 0.250966 | 1 |
| CA1-body (lh) | Gaussian | 1.07605E-06 | 9.07644E-06 | 0.118554 | 0.906418685 | 0.164168 | 1 |
| subiculum-head (lh) | Gaussian | 1.03543E-05 | 9.38928E-06 | 1.102778 | 0.278891688 | 0.225853 | 1 |
| hippocampal-fissure (lh) | Gaussian | 1.67491E-05 | 7.24574E-06 | 2.311585 | 0.027850386 | 0.474436 | 1 |
| presubiculum-head (lh) | Gaussian | 6.81506E-06 | 5.41065E-06 | 1.259564 | 0.21753835 | 0.260388 | 1 |
| CA1-head (lh) | Gaussian | 1.37312E-05 | 2.2338E-05 | 0.614701 | 0.543386494 | 0.169671 | 1 |
| presubiculum-body (lh) | Gaussian | 2.89293E-05 | 5.82256E-06 | 4.968488 | 2.54643E-05 | 0.333175 | 0.005424 |
| parasubiculum (lh) | Gaussian | 9.74153E-07 | 3.51645E-06 | 0.277027 | 0.783659139 | 0.624993 | 1 |
| molecular_layer_HP-head (lh) | Gaussian | 1.32497E-05 | 6.69809E-06 | 1.978135 | 0.057161265 | 0.712899 | 1 |
| molecular_layer_HP-body (lh) | Gaussian | -9.95092E-06 | 4.93205E-06 | -2.0176 | 0.052655378 | 0.898284 | 1 |
| GC-ML-DG-head (lh) | Gaussian | 3.3518E-06 | 7.08183E-06 | 0.473296 | 0.639427626 | 0.162226 | 1 |
| CA3-body (lh) | Gaussian | 5.83264E-06 | 5.82798E-06 | 1.0008 | 0.324927901 | 0.359833 | 1 |
| GC-ML-DG-body (lh) | Gaussian | 8.30185E-06 | 5.11164E-06 | 1.624107 | 0.114815149 | 0.840816 | 1 |
| CA4-head (lh) | Gaussian | 1.74913E-06 | 6.12934E-06 | 0.28537 | 0.777321896 | 0.20852 | 1 |
| CA4-body (lh) | Gaussian | 5.9481E-06 | 5.00542E-06 | 1.188332 | 0.244025246 | 0.627759 | 1 |
| fimbria (lh) | Gaussian | 5.12819E-06 | 4.01276E-06 | 1.277973 | 0.211059429 | 0.580039 | 1 |
| CA3-head (lh) | Gaussian | 1.07942E-05 | 6.29519E-06 | 1.714679 | 0.09671854 | 0.788484 | 1 |
| HATA (lh) | Gaussian | 3.62057E-06 | 2.00092E-06 | 1.809451 | 0.080411483 | 0.536865 | 1 |
| Whole_hippocampal_body (lh) | Gaussian | 6.1205E-05 | 2.80549E-05 | 2.181618 | 0.037106246 | 0.728755 | 1 |
| Whole_hippocampal_head (lh) | Gaussian | 6.46402E-05 | 5.25943E-05 | 1.229033 | 0.228612731 | 0.234329 | 1 |
| Whole_hippocampus (lh) | Gaussian | 0.000163905 | 8.73079E-05 | 1.87732 | 0.070228538 | 0.248111 | 1 |
| Hippocampal_tail (rh) | Gaussian | 8.79883E-05 | 2.4165E-05 | 3.641142 | 0.001013023 | 0.481776 | 0.215774 |
| subiculum-body (rh) | Gaussian | 1.92573E-05 | 7.96549E-06 | 2.417585 | 0.021908486 | 0.376352 | 1 |
| CA1-body (rh) | Gaussian | 3.89989E-06 | 8.26343E-06 | 0.471946 | 0.640380408 | 0.488107 | 1 |
| subiculum-head (rh) | Gaussian | 1.86294E-05 | 8.87891E-06 | 2.098164 | 0.044416108 | 0.005637 | 1 |
| hippocampal-fissure (rh) | Gaussian | 2.29451E-05 | 6.39306E-06 | 3.589069 | 0.001164755 | 0.573941 | 0.248093 |
| presubiculum-head (rh) | Gaussian | 1.15094E-05 | 5.28406E-06 | 2.178133 | 0.037388562 | 0.980471 | 1 |
| CA1-head (rh) | Gaussian | -1.65375E-05 | 2.06175E-05 | -0.80211 | 0.428798689 | 0.409356 | 1 |
| presubiculum-body (rh) | Gaussian | 1.79943E-05 | 6.41204E-06 | 2.806326 | 0.008717141 | 0.653893 | 1 |
| parasubiculum (rh) | Gaussian | 4.13949E-06 | 3.40657E-06 | 1.21515 | 0.233785929 | 0.470646 | 1 |
| molecular_layer_HP-head (rh) | Gaussian | 9.51112E-06 | 1.00611E-05 | 0.94534 | 0.352037732 | 0.831733 | 1 |
| molecular_layer_HP-body (rh) | Gaussian | -1.99008E-05 | 8.07119E-06 | -2.46565 | 0.01961645 | 0.298668 | 1 |
| GC-ML-DG-head (rh) | Gaussian | -4.83242E-07 | 6.71722E-06 | -0.07194 | 0.94312651 | 0.906691 | 1 |
| CA3-body (rh) | Gaussian | 1.68368E-05 | 5.55864E-06 | 3.028939 | 0.005010855 | 0.710568 | 1 |
| GC-ML-DG-body (rh) | Gaussian | 1.76475E-05 | 5.29221E-06 | 3.334624 | 0.002283382 | 0.034138 | 0.48636 |
| CA4-head (rh) | Gaussian | -1.61505E-06 | 5.99018E-06 | -0.26962 | 0.789301616 | 0.633562 | 1 |
| CA4-body (rh) | Gaussian | 1.45861E-05 | 4.57498E-06 | 3.188238 | 0.003338212 | 0.201064 | 0.711039 |
| fimbria (rh) | Gaussian | -2.98889E-06 | 4.43151E-06 | -0.67446 | 0.505181559 | 0.786313 | 1 |
| CA3-head (rh) | Gaussian | 8.83275E-06 | 5.46608E-06 | 1.615921 | 0.116580647 | 0.106828 | 1 |
| HATA (rh) | Gaussian | 7.19382E-06 | 3.07939E-06 | 2.336114 | 0.026358228 | 0.71175 | 1 |
| Whole_hippocampal_body (rh) | Gaussian | 6.73322E-05 | 3.16175E-05 | 2.129588 | 0.041525599 | 0.287669 | 1 |
| Whole_hippocampal_head (rh) | Gaussian | 4.11802E-05 | 5.8212E-05 | 0.707418 | 0.484766481 | 0.441086 | 1 |
| Whole_hippocampus (rh) | Gaussian | 0.000196501 | 0.000100849 | 1.948466 | 0.060766919 | 0.366972 | 1 |
| AV (lh) | Gaussian | -8.9303E-06 | 5.11403E-06 | -1.74623 | 0.091004262 | 0.884798 | 1 |
| CeM (lh) | Gaussian | -1.05373E-05 | 3.51607E-06 | -2.9969 | 0.005432101 | 0.58012 | 1 |
| CL (lh) | Gaussian | -8.41201E-06 | 2.1213E-06 | -3.9655 | 0.000419791 | 0.187694 | 0.089415 |
| CM (lh) | Gaussian | -1.35716E-05 | 7.91735E-06 | -1.71416 | 0.096815758 | 0.75699 | 1 |
| LD (lh) | Gaussian | -6.80393E-06 | 1.80764E-06 | -3.76398 | 0.000727255 | 0.531155 | 0.154905 |
| LGN (lh) | Gaussian | 1.12922E-05 | 1.29834E-05 | 0.869746 | 0.391345337 | 0.779313 | 1 |
| LP (lh) | Gaussian | -1.49211E-05 | 5.05147E-06 | -2.9538 | 0.006051989 | 0.676607 | 1 |
| L-Sg (lh) | Gaussian | -2.74667E-06 | 2.64138E-06 | -1.03986 | 0.306713259 | 0.511646 | 1 |
| MDl (lh) | Gaussian | -6.82537E-06 | 8.70751E-06 | -0.78385 | 0.439274138 | 0.342244 | 1 |
| MDm (lh) | Gaussian | -8.25888E-06 | 2.05908E-05 | -0.4011 | 0.69119259 | 0.105132 | 1 |
| MGN (lh) | Gaussian | -7.5149E-07 | 8.17907E-06 | -0.09188 | 0.927404516 | 0.094708 | 1 |
| MV(Re) (lh) | Gaussian | -2.45761E-06 | 1.36384E-06 | -1.80198 | 0.081606613 | 0.757716 | 1 |
| Pc (lh) | Gaussian | -2.15345E-07 | 1.15553E-07 | -1.86361 | 0.072190872 | 0.137617 | 1 |
| Pf (lh) | Gaussian | -5.14567E-06 | 1.83274E-06 | -2.80764 | 0.008689227 | 0.912481 | 1 |
| Pt (lh) | Gaussian | -6.05857E-07 | 2.11459E-07 | -2.86513 | 0.007543695 | 0.367295 | 1 |
| PuA (lh) | Gaussian | 1.51221E-05 | 6.91816E-06 | 2.185852 | 0.036765863 | 0.596392 | 1 |
| PuI (lh) | Gaussian | 5.6335E-05 | 2.32297E-05 | 2.425129 | 0.021533293 | 0.048819 | 1 |
| PuL (lh) | Gaussian | 5.79785E-05 | 1.80228E-05 | 3.21695 | 0.003100046 | 0.533994 | 0.66031 |
| PuM (lh) | Gaussian | 0.000114645 | 5.33562E-05 | 2.148667 | 0.039853421 | 0.791834 | 1 |
| VA (lh) | Gaussian | 1.75403E-06 | 1.41266E-05 | 0.124165 | 0.902012741 | 0.855398 | 1 |
| VAmc (lh) | Gaussian | -1.71794E-06 | 9.9023E-07 | -1.73489 | 0.093024373 | 0.801687 | 1 |
| VLa (lh) | Gaussian | -1.64203E-05 | 1.8282E-05 | -0.89817 | 0.376248171 | 0.021289 | 1 |
| VLp (lh) | Gaussian | -2.16502E-05 | 2.50913E-05 | -0.86286 | 0.395062515 | 0.142552 | 1 |
| VM (lh) | Gaussian | 1.61113E-07 | 7.46253E-07 | 0.215895 | 0.830530448 | 0.078189 | 1 |
| VPL (lh) | Gaussian | 7.04435E-07 | 2.49828E-05 | 0.028197 | 0.977691929 | 0.234847 | 1 |
| Whole_thalamus (lh) | Gaussian | 0.000128021 | 0.000165787 | 0.772199 | 0.446037713 | 0.159525 | 1 |
| AV (rh) | Gaussian | -1.2746E-05 | 5.54815E-06 | -2.29734 | 0.028751905 | 0.159843 | 1 |
| CeM (rh) | Gaussian | -1.18202E-05 | 4.61753E-06 | -2.55986 | 0.015750034 | 0.873961 | 1 |
| CL (rh) | Gaussian | -9.08853E-06 | 2.57574E-06 | -3.52851 | 0.001369019 | 0.798553 | 0.291601 |
| CM (rh) | Gaussian | -1.49741E-05 | 7.58932E-06 | -1.97305 | 0.057765257 | 0.029991 | 1 |
| LD (rh) | Gaussian | -8.74674E-06 | 2.45211E-06 | -3.56703 | 0.001235411 | 0.989011 | 0.263142 |
| LGN (rh) | Gaussian | 1.51967E-05 | 1.25249E-05 | 1.213317 | 0.234475711 | 0.042442 | 1 |
| LP (rh) | Gaussian | -8.43066E-06 | 5.88314E-06 | -1.43302 | 0.162191444 | 0.33173 | 1 |
| L-Sg (rh) | Gaussian | -1.21998E-06 | 1.82913E-06 | -0.66697 | 0.509888785 | 0.001709 | 1 |
| MDl (rh) | Gaussian | -4.5286E-05 | 1.15949E-05 | -3.90568 | 0.000494515 | 0.02932 | 0.105332 |
| MDm (rh) | Gaussian | -0.00010193 | 3.05902E-05 | -3.33212 | 0.002298344 | 0.049509 | 0.489547 |
| MGN (rh) | Gaussian | 5.1707E-06 | 4.76237E-06 | 1.085741 | 0.28624136 | 0.608256 | 1 |
| MV(Re) (rh) | Gaussian | -3.62942E-06 | 1.81446E-06 | -2.00027 | 0.054594172 | 0.975214 | 1 |
| Pc (rh) | Gaussian | 6.67851E-08 | 1.58417E-07 | 0.421577 | 0.676339414 | 0.007489 | 1 |
| Pf (rh) | Gaussian | -6.00094E-06 | 1.7994E-06 | -3.33496 | 0.002281379 | 0.327382 | 0.485934 |
| Pt (rh) | Gaussian | -8.17714E-07 | 2.31587E-07 | -3.53091 | 0.001360309 | 0.224531 | 0.289746 |
| PuA (rh) | Gaussian | -5.03557E-06 | 6.52853E-06 | -0.77132 | 0.446552072 | 0.468341 | 1 |
| PuI (rh) | Gaussian | 2.33231E-05 | 9.06017E-06 | 2.574248 | 0.015225747 | 0.370423 | 1 |
| PuL (rh) | Gaussian | 9.54607E-06 | 1.16138E-05 | 0.821959 | 0.417585362 | 0.144858 | 1 |
| PuM (rh) | Gaussian | 3.67879E-05 | 3.51511E-05 | 1.046564 | 0.303660064 | 0.991179 | 1 |
| VA (rh) | Gaussian | -2.77259E-05 | 1.34408E-05 | -2.06282 | 0.047879525 | 0.375773 | 1 |
| VAmc (rh) | Gaussian | -3.02275E-06 | 1.00624E-06 | -3.00402 | 0.005335781 | 0.486363 | 1 |
| VLa (rh) | Gaussian | -2.25866E-05 | 1.93351E-05 | -1.16817 | 0.251939399 | 0.029667 | 1 |
| VLp (rh) | Gaussian | -1.60916E-05 | 2.60782E-05 | -0.61705 | 0.541855623 | 0.035901 | 1 |
| VM (rh) | Gaussian | -9.55461E-08 | 6.42124E-07 | -0.1488 | 0.882709672 | 0.050822 | 1 |
| VPL (rh) | Gaussian | 5.17271E-06 | 2.42623E-05 | 0.213199 | 0.832613729 | 0.032383 | 1 |
| Whole_thalamus (rh) | Gaussian | -0.000203985 | 0.000159342 | -1.28017 | 0.210295106 | 0.016407 | 1 |

GLM analysis of epilepsy with a right-sided focus compared to healthy controls, using age and gender as covariates

| **Region** | **Method** | **Beta** | **SE** | **t_value** | **p_value** | **resid_sw_p** | **adj_p_bonferroni** |
| --- | --- | --- | --- | --- | --- | --- | --- |
| Left-Cerebral-White-Matter () | Gaussian | -0.007999539 | 0.003337074 | -2.39717 | 0.022953956 | 0.112433 | 1 |
| Left-Lateral-Ventricle () | Gaussian | -0.000978739 | 0.001295853 | -0.75529 | 0.455966959 | 0.002796 | 1 |
| Left-Inf-Lat-Vent () | Gaussian | -5.84157E-05 | 5.18413E-05 | -1.12682 | 0.268752314 | 0.15883 | 1 |
| Left-Cerebellum-White-Matter () | Gaussian | 0.001594106 | 0.000663706 | 2.401826 | 0.022711632 | 0.693887 | 1 |
| Left-Cerebellum-Cortex () | Gaussian | 0.002817625 | 0.001569705 | 1.795002 | 0.082735213 | 0.524781 | 1 |
| Left-Thalamus () | Gaussian | -5.59569E-05 | 0.000122805 | -0.45566 | 0.651917335 | 0.375698 | 1 |
| Left-Caudate () | Gaussian | 9.98001E-05 | 0.000105601 | 0.945064 | 0.352176202 | 0.863212 | 1 |
| Left-Putamen () | Gaussian | 0.000148768 | 0.000154071 | 0.965584 | 0.341972182 | 0.573471 | 1 |
| Left-Pallidum () | Gaussian | -0.000135454 | 5.35776E-05 | -2.52819 | 0.016963571 | 0.234389 | 1 |
| Left-Hippocampus () | Gaussian | 0.000243815 | 0.000121054 | 2.014097 | 0.053042639 | 0.381151 | 1 |
| Left-Amygdala () | Gaussian | 0.00024248 | 4.93097E-05 | 4.917498 | 2.94059E-05 | 0.973285 | 0.006263 |
| Left-Accumbens-area () | Gaussian | 1.90959E-05 | 2.19191E-05 | 0.871199 | 0.390564392 | 0.058439 | 1 |
| Left-VentralDC () | Gaussian | 2.92655E-05 | 0.000109441 | 0.26741 | 0.790983625 | 0.474521 | 1 |
| Left-choroid-plexus () | Gaussian | 3.59925E-05 | 5.91639E-05 | 0.608352 | 0.547532994 | 0.860458 | 1 |
| Right-Cerebral-White-Matter () | Gaussian | -0.00608203 | 0.003507639 | -1.73394 | 0.093196028 | 0.332135 | 1 |
| Right-Lateral-Ventricle () | Gaussian | -0.000722169 | 0.001209 | -0.59733 | 0.554770705 | 0.001073 | 1 |
| Right-Inf-Lat-Vent () | Gaussian | -9.53511E-06 | 7.86927E-05 | -0.12117 | 0.904365164 | 0.000249 | 1 |
| Right-Cerebellum-White-Matter () | Gaussian | 0.001084311 | 0.000489925 | 2.213216 | 0.034632265 | 0.605215 | 1 |
| Right-Cerebellum-Cortex () | Gaussian | 0.003194659 | 0.001553047 | 2.057026 | 0.048468907 | 0.913628 | 1 |
| Right-Thalamus () | Gaussian | -0.000179907 | 0.000136246 | -1.32046 | 0.196668426 | 0.257732 | 1 |
| Right-Caudate () | Gaussian | -0.000140437 | 0.000142555 | -0.98514 | 0.332432808 | 0.006518 | 1 |
| Right-Putamen () | Gaussian | 0.000160287 | 0.000158146 | 1.013537 | 0.318908723 | 0.770789 | 1 |
| Right-Pallidum () | Gaussian | 7.68964E-05 | 5.29852E-05 | 1.451281 | 0.157079247 | 0.07145 | 1 |
| Right-Hippocampus () | Gaussian | 0.000326104 | 0.000112209 | 2.906225 | 0.006813789 | 0.739929 | 1 |
| Right-Amygdala () | Gaussian | 0.000340675 | 5.544E-05 | 6.14493 | 9.30496E-07 | 0.985119 | 0.000198 |
| Right-Accumbens-area () | Gaussian | 3.61133E-05 | 3.5069E-05 | 1.02978 | 0.31134451 | 0.06292 | 1 |
| Right-VentralDC () | Gaussian | -0.000101314 | 0.000113936 | -0.88922 | 0.380961403 | 0.33231 | 1 |
| Right-choroid-plexus () | Gaussian | -9.04115E-06 | 5.5781E-05 | -0.16208 | 0.872327338 | 0.729038 | 1 |
| Brain-Stem () | Gaussian | 0.001595633 | 0.000592751 | 2.691912 | 0.011506194 | 0.507593 | 1 |
| CSF () | Gaussian | 8.00678E-05 | 7.43852E-05 | 1.076394 | 0.29033146 | 0.688639 | 1 |
| ctx-lh-caudalanteriorcingulate () | Gaussian | -0.000334503 | 0.000131227 | -2.54905 | 0.016155115 | 0.76845 | 1 |
| ctx-lh-caudalmiddlefrontal () | Gaussian | -0.00051758 | 0.000352044 | -1.47021 | 0.151915129 | 0.216103 | 1 |
| ctx-lh-cuneus () | Gaussian | 3.54843E-05 | 0.000278042 | 0.127622 | 0.899299754 | 0.97923 | 1 |
| ctx-lh-entorhinal () | Gaussian | 0.0005032 | 9.21866E-05 | 5.458495 | 6.38362E-06 | 0.068738 | 0.00136 |
| ctx-lh-fusiform () | Gaussian | 0.00132973 | 0.000290577 | 4.576179 | 7.68804E-05 | 0.093907 | 0.016376 |
| ctx-lh-inferiorparietal () | Gaussian | -0.000178912 | 0.000361707 | -0.49463 | 0.624462347 | 0.488084 | 1 |
| ctx-lh-inferiortemporal () | Gaussian | 0.002065811 | 0.000380525 | 5.428839 | 6.94061E-06 | 0.580336 | 0.001478 |
| ctx-lh-isthmuscingulate () | Gaussian | 1.02806E-05 | 0.000103915 | 0.098933 | 0.921849743 | 0.519413 | 1 |
| ctx-lh-lateraloccipital () | Gaussian | -0.00023177 | 0.00048002 | -0.48283 | 0.632717772 | 0.178557 | 1 |
| ctx-lh-lateralorbitofrontal () | Gaussian | 0.000843682 | 0.000241297 | 3.496452 | 0.001490782 | 0.968266 | 0.317537 |
| ctx-lh-lingual () | Gaussian | 0.000354294 | 0.0002822 | 1.255471 | 0.21899903 | 0.647891 | 1 |
| ctx-lh-medialorbitofrontal () | Gaussian | 0.000691029 | 0.000142374 | 4.853616 | 3.52128E-05 | 0.143791 | 0.0075 |
| ctx-lh-middletemporal () | Gaussian | 5.04896E-05 | 0.000464577 | 0.108679 | 0.914181059 | 0.705611 | 1 |
| ctx-lh-parahippocampal () | Gaussian | 0.000398327 | 0.000100595 | 3.959724 | 0.00042649 | 0.212072 | 0.090842 |
| ctx-lh-paracentral () | Gaussian | -0.000238919 | 0.000188159 | -1.26977 | 0.213927936 | 0.218749 | 1 |
| ctx-lh-parsopercularis () | Gaussian | -0.000144837 | 0.000240775 | -0.60154 | 0.551998055 | 0.797604 | 1 |
| ctx-lh-parsorbitalis () | Gaussian | -0.000206866 | 0.000120693 | -1.71398 | 0.096848586 | 0.869126 | 1 |
| ctx-lh-parstriangularis () | Gaussian | -0.000163467 | 0.000306978 | -0.5325 | 0.598300287 | 0.51762 | 1 |
| ctx-lh-pericalcarine () | Gaussian | -0.000166474 | 0.00014284 | -1.16545 | 0.253018376 | 0.01573 | 1 |
| ctx-lh-postcentral () | Gaussian | -0.000553156 | 0.000371184 | -1.49025 | 0.146598595 | 0.507984 | 1 |
| ctx-lh-posteriorcingulate () | Gaussian | -0.000142144 | 0.000144463 | -0.98395 | 0.333010654 | 0.357266 | 1 |
| ctx-lh-precentral () | Gaussian | -0.000320358 | 0.000432978 | -0.73989 | 0.465114972 | 0.444443 | 1 |
| ctx-lh-precuneus () | Gaussian | -0.000267973 | 0.000331515 | -0.80833 | 0.42526501 | 0.867455 | 1 |
| ctx-lh-rostralanteriorcingulate () | Gaussian | -0.000107107 | 0.000185058 | -0.57878 | 0.567060735 | 0.747599 | 1 |
| ctx-lh-rostralmiddlefrontal () | Gaussian | -0.001283062 | 0.000600429 | -2.13691 | 0.040876681 | 0.047918 | 1 |
| ctx-lh-superiorfrontal () | Gaussian | -0.001269983 | 0.000683528 | -1.85798 | 0.073010297 | 0.887418 | 1 |
| ctx-lh-superiorparietal () | Gaussian | -0.000263164 | 0.000427034 | -0.61626 | 0.542370685 | 0.651938 | 1 |
| ctx-lh-superiortemporal () | Gaussian | 0.001877847 | 0.000387273 | 4.848893 | 3.56849E-05 | 0.253836 | 0.007601 |
| ctx-lh-supramarginal () | Gaussian | -0.000387882 | 0.000360692 | -1.07538 | 0.290775433 | 0.509826 | 1 |
| ctx-lh-transversetemporal () | Gaussian | 4.493E-05 | 5.93689E-05 | 0.756792 | 0.455077192 | 0.271159 | 1 |
| ctx-lh-insula () | Gaussian | 0.00020798 | 0.000151646 | 1.371478 | 0.180397672 | 0.4134 | 1 |
| ctx-rh-caudalanteriorcingulate () | Gaussian | -0.000103273 | 0.000141148 | -0.73167 | 0.470047389 | 0.334031 | 1 |
| ctx-rh-caudalmiddlefrontal () | Gaussian | -0.000596338 | 0.00037867 | -1.57482 | 0.125785337 | 0.299471 | 1 |
| ctx-rh-cuneus () | Gaussian | -8.74541E-05 | 0.000206366 | -0.42378 | 0.674747792 | 0.219509 | 1 |
| ctx-rh-entorhinal () | Gaussian | 0.000549529 | 0.000108477 | 5.06587 | 1.93424E-05 | 0.08069 | 0.00412 |
| ctx-rh-fusiform () | Gaussian | 0.000825131 | 0.000248736 | 3.3173 | 0.002389072 | 0.296712 | 0.508872 |
| ctx-rh-inferiorparietal () | Gaussian | -0.000529675 | 0.000530398 | -0.99864 | 0.325958524 | 0.299642 | 1 |
| ctx-rh-inferiortemporal () | Gaussian | 0.001188666 | 0.000396414 | 2.99855 | 0.005409659 | 0.718799 | 1 |
| ctx-rh-isthmuscingulate () | Gaussian | -8.97204E-05 | 0.000118159 | -0.75932 | 0.453589082 | 0.261015 | 1 |
| ctx-rh-lateraloccipital () | Gaussian | 0.00068476 | 0.000414468 | 1.652144 | 0.108935366 | 0.749347 | 1 |
| ctx-rh-lateralorbitofrontal () | Gaussian | 0.001748335 | 0.000248246 | 7.042742 | 7.90145E-08 | 0.922657 | 1.68E-05 |
| ctx-rh-lingual () | Gaussian | 0.000614687 | 0.000262808 | 2.338916 | 0.026192483 | 0.378513 | 1 |
| ctx-rh-medialorbitofrontal () | Gaussian | 0.000938431 | 0.000154483 | 6.074658 | 1.13187E-06 | 0.769874 | 0.000241 |
| ctx-rh-middletemporal () | Gaussian | -0.000129886 | 0.000325269 | -0.39932 | 0.692486734 | 0.292475 | 1 |
| ctx-rh-parahippocampal () | Gaussian | 0.000435056 | 7.88054E-05 | 5.520641 | 5.35759E-06 | 0.900312 | 0.001141 |
| ctx-rh-paracentral () | Gaussian | -0.000439444 | 0.000174376 | -2.5201 | 0.017287013 | 0.516318 | 1 |
| ctx-rh-parsopercularis () | Gaussian | -0.00016276 | 0.000162218 | -1.00335 | 0.323718838 | 0.77437 | 1 |
| ctx-rh-parsorbitalis () | Gaussian | 0.000350429 | 0.00010128 | 3.46 | 0.001641968 | 0.308901 | 0.349739 |
| ctx-rh-parstriangularis () | Gaussian | -0.000174243 | 0.000286228 | -0.60876 | 0.547267025 | 0.259704 | 1 |
| ctx-rh-pericalcarine () | Gaussian | -0.000302074 | 0.000171674 | -1.75958 | 0.088674506 | 0.94418 | 1 |
| ctx-rh-postcentral () | Gaussian | 0.000153843 | 0.000384898 | 0.399699 | 0.692209533 | 0.417243 | 1 |
| ctx-rh-posteriorcingulate () | Gaussian | -6.73379E-05 | 0.000184375 | -0.36522 | 0.717508114 | 0.425414 | 1 |
| ctx-rh-precentral () | Gaussian | -0.000168786 | 0.000401234 | -0.42067 | 0.676996773 | 0.394054 | 1 |
| ctx-rh-precuneus () | Gaussian | -8.50695E-05 | 0.00028174 | -0.30194 | 0.764778959 | 0.15313 | 1 |
| ctx-rh-rostralanteriorcingulate () | Gaussian | 0.000199409 | 0.000146628 | 1.359964 | 0.183974515 | 0.354471 | 1 |
| ctx-rh-rostralmiddlefrontal () | Gaussian | -0.001446657 | 0.00055328 | -2.61469 | 0.013836949 | 0.966763 | 1 |
| ctx-rh-superiorfrontal () | Gaussian | -0.000604973 | 0.000867714 | -0.6972 | 0.491043612 | 0.299069 | 1 |
| ctx-rh-superiorparietal () | Gaussian | -0.00070333 | 0.000458918 | -1.53258 | 0.13585772 | 0.620388 | 1 |
| ctx-rh-superiortemporal () | Gaussian | 0.002148162 | 0.000394459 | 5.445843 | 6.61554E-06 | 0.6501 | 0.001409 |
| ctx-rh-supramarginal () | Gaussian | -0.000337109 | 0.000337004 | -1.00031 | 0.325160269 | 0.091061 | 1 |
| ctx-rh-transversetemporal () | Gaussian | 0.000112546 | 4.85986E-05 | 2.315826 | 0.027587033 | 0.41237 | 1 |
| ctx-rh-insula () | Gaussian | 0.000324108 | 0.000172132 | 1.8829 | 0.069443112 | 0.792022 | 1 |
| Medulla () | Gaussian | 0.000121671 | 0.000154041 | 0.789857 | 0.435810431 | 0.773076 | 1 |
| Pons () | Gaussian | 0.001454832 | 0.000550753 | 2.641534 | 0.012981148 | 0.959101 | 1 |
| SCP () | Gaussian | -3.39843E-05 | 1.93911E-05 | -1.75258 | 0.089890627 | 0.341125 | 1 |
| Midbrain () | Gaussian | 0.000521435 | 0.000261511 | 1.993934 | 0.055318556 | 0.7285 | 1 |
| Whole_brainstem () | Gaussian | 0.002063954 | 0.000923147 | 2.235781 | 0.032957144 | 0.275339 | 1 |
| Lateral-nucleus (lh) | Gaussian | 4.90385E-05 | 2.59353E-05 | 1.890798 | 0.068344226 | 0.544058 | 1 |
| Basal-nucleus (lh) | Gaussian | 2.95476E-05 | 1.52036E-05 | 1.943456 | 0.061394881 | 0.887328 | 1 |
| Accessory-Basal-nucleus (lh) | Gaussian | 2.3703E-05 | 8.6138E-06 | 2.751751 | 0.009957491 | 0.427288 | 1 |
| Anterior-amygdaloid-area-AAA (lh) | Gaussian | 1.31874E-06 | 3.13883E-06 | 0.420137 | 0.677379305 | 0.322819 | 1 |
| Central-nucleus (lh) | Gaussian | 4.62326E-06 | 1.7533E-06 | 2.636889 | 0.013125645 | 0.931926 | 1 |
| Medial-nucleus (lh) | Gaussian | 3.679E-06 | 1.5288E-06 | 2.406458 | 0.022472839 | 0.290471 | 1 |
| Cortical-nucleus (lh) | Gaussian | 2.51948E-06 | 1.10212E-06 | 2.286041 | 0.029485191 | 0.263151 | 1 |
| Corticoamygdaloid-transitio (lh) | Gaussian | 9.0371E-06 | 5.0916E-06 | 1.774902 | 0.086062551 | 0.121855 | 1 |
| Paralaminar-nucleus (lh) | Gaussian | 2.16025E-06 | 1.72414E-06 | 1.252942 | 0.219905079 | 0.471429 | 1 |
| Whole_amygdala (lh) | Gaussian | 0.000125627 | 5.87939E-05 | 2.136735 | 0.040892011 | 0.204979 | 1 |
| Lateral-nucleus (rh) | Gaussian | -5.09679E-06 | 3.53249E-05 | -0.14428 | 0.886241844 | 0.651965 | 1 |
| Basal-nucleus (rh) | Gaussian | 3.70173E-05 | 1.36737E-05 | 2.707191 | 0.011090675 | 0.451386 | 1 |
| Accessory-Basal-nucleus (rh) | Gaussian | 2.46268E-05 | 7.95084E-06 | 3.097378 | 0.004212523 | 0.973393 | 0.897267 |
| Anterior-amygdaloid-area-AAA (rh) | Gaussian | 1.11322E-06 | 2.73245E-06 | 0.407408 | 0.6866012 | 0.700223 | 1 |
| Central-nucleus (rh) | Gaussian | 3.20469E-06 | 2.06819E-06 | 1.549515 | 0.131744126 | 0.195351 | 1 |
| Medial-nucleus (rh) | Gaussian | 2.52682E-06 | 1.84426E-06 | 1.370099 | 0.1808231 | 0.562788 | 1 |
| Cortical-nucleus (rh) | Gaussian | 2.73758E-06 | 1.16363E-06 | 2.352633 | 0.025394623 | 0.748081 | 1 |
| Corticoamygdaloid-transitio (rh) | Gaussian | 4.02494E-06 | 6.43514E-06 | 0.625463 | 0.536396678 | 0.931353 | 1 |
| Paralaminar-nucleus (rh) | Gaussian | 3.98365E-06 | 1.64409E-06 | 2.423011 | 0.021637999 | 0.29515 | 1 |
| Whole_amygdala (rh) | Gaussian | 7.41382E-05 | 6.22707E-05 | 1.19058 | 0.243154283 | 0.536072 | 1 |
| Hippocampal_tail (lh) | Gaussian | 4.2065E-05 | 2.00768E-05 | 2.095211 | 0.044696767 | 0.937971 | 1 |
| subiculum-body (lh) | Gaussian | 1.52437E-05 | 6.64757E-06 | 2.29312 | 0.029023649 | 0.304522 | 1 |
| CA1-body (lh) | Gaussian | 9.78592E-06 | 8.74177E-06 | 1.119444 | 0.271833823 | 0.522218 | 1 |
| subiculum-head (lh) | Gaussian | -3.02269E-06 | 7.85751E-06 | -0.38469 | 0.703182346 | 0.103701 | 1 |
| hippocampal-fissure (lh) | Gaussian | 3.08294E-05 | 6.6653E-06 | 4.62536 | 6.6961E-05 | 0.020784 | 0.014263 |
| presubiculum-head (lh) | Gaussian | 5.48442E-06 | 4.98021E-06 | 1.101244 | 0.279548197 | 0.043474 | 1 |
| CA1-head (lh) | Gaussian | 3.14126E-05 | 2.37183E-05 | 1.324402 | 0.195371222 | 0.083159 | 1 |
| presubiculum-body (lh) | Gaussian | 2.30267E-05 | 5.66769E-06 | 4.06279 | 0.000321234 | 0.979718 | 0.068423 |
| parasubiculum (lh) | Gaussian | 3.99513E-06 | 3.49774E-06 | 1.142204 | 0.262404049 | 0.277156 | 1 |
| molecular_layer_HP-head (lh) | Gaussian | 7.03286E-06 | 5.72636E-06 | 1.228155 | 0.228937453 | 0.691419 | 1 |
| molecular_layer_HP-body (lh) | Gaussian | -1.78716E-05 | 5.0257E-06 | -3.55604 | 0.001272188 | 0.670372 | 0.270976 |
| GC-ML-DG-head (lh) | Gaussian | 1.73042E-05 | 7.73219E-06 | 2.237946 | 0.032800296 | 0.117311 | 1 |
| CA3-body (lh) | Gaussian | 1.55757E-05 | 6.299E-06 | 2.472729 | 0.019298216 | 0.452287 | 1 |
| GC-ML-DG-body (lh) | Gaussian | 1.12574E-05 | 4.67317E-06 | 2.408942 | 0.022345703 | 0.26518 | 1 |
| CA4-head (lh) | Gaussian | 1.43799E-05 | 6.73941E-06 | 2.133705 | 0.041159565 | 0.420493 | 1 |
| CA4-body (lh) | Gaussian | 1.04223E-05 | 4.53044E-06 | 2.300494 | 0.028549869 | 0.211058 | 1 |
| fimbria (lh) | Gaussian | -9.99431E-06 | 3.9231E-06 | -2.54755 | 0.016211799 | 0.526723 | 1 |
| CA3-head (lh) | Gaussian | 1.93046E-05 | 6.51599E-06 | 2.962649 | 0.00591955 | 0.732532 | 1 |
| HATA (lh) | Gaussian | 9.30925E-06 | 2.34274E-06 | 3.973658 | 0.000410494 | 0.041226 | 0.087435 |
| Whole_hippocampal_body (lh) | Gaussian | 5.74457E-05 | 2.50731E-05 | 2.291127 | 0.029152944 | 0.189507 | 1 |
| Whole_hippocampal_head (lh) | Gaussian | 0.0001052 | 5.61499E-05 | 1.873563 | 0.070761841 | 0.170354 | 1 |
| Whole_hippocampus (lh) | Gaussian | 0.000204711 | 9.03164E-05 | 2.266598 | 0.03078714 | 0.137657 | 1 |
| Hippocampal_tail (rh) | Gaussian | 0.000110995 | 2.38567E-05 | 4.652541 | 6.20353E-05 | 0.150793 | 0.013214 |
| subiculum-body (rh) | Gaussian | 3.73092E-05 | 7.37082E-06 | 5.061745 | 1.95691E-05 | 0.365135 | 0.004168 |
| CA1-body (rh) | Gaussian | 2.12479E-05 | 8.85849E-06 | 2.398591 | 0.022879825 | 0.581924 | 1 |
| subiculum-head (rh) | Gaussian | 6.88877E-06 | 8.82297E-06 | 0.780776 | 0.441051825 | 0.001975 | 1 |
| hippocampal-fissure (rh) | Gaussian | 4.34133E-05 | 5.98983E-06 | 7.247832 | 4.54953E-08 | 0.880897 | 9.69E-06 |
| presubiculum-head (rh) | Gaussian | 1.01315E-05 | 5.0025E-06 | 2.025281 | 0.051815966 | 0.996178 | 1 |
| CA1-head (rh) | Gaussian | 1.27678E-06 | 2.25322E-05 | 0.056665 | 0.955188087 | 0.343029 | 1 |
| presubiculum-body (rh) | Gaussian | 2.69162E-05 | 4.53604E-06 | 5.933852 | 1.67768E-06 | 0.892648 | 0.000357 |
| parasubiculum (rh) | Gaussian | 5.06683E-07 | 3.02245E-06 | 0.16764 | 0.867991764 | 0.946848 | 1 |
| molecular_layer_HP-head (rh) | Gaussian | 1.16557E-05 | 8.00866E-06 | 1.455389 | 0.155946963 | 0.654359 | 1 |
| molecular_layer_HP-body (rh) | Gaussian | -2.71278E-05 | 8.26328E-06 | -3.28294 | 0.002612799 | 0.338347 | 0.556526 |
| GC-ML-DG-head (rh) | Gaussian | 1.16911E-05 | 6.78646E-06 | 1.722711 | 0.095236207 | 0.946312 | 1 |
| CA3-body (rh) | Gaussian | 2.30699E-05 | 5.85135E-06 | 3.942665 | 0.000446905 | 0.474466 | 0.095191 |
| GC-ML-DG-body (rh) | Gaussian | 2.13473E-05 | 4.53876E-06 | 4.703332 | 5.3776E-05 | 0.017553 | 0.011454 |
| CA4-head (rh) | Gaussian | 1.07799E-05 | 5.96405E-06 | 1.807476 | 0.080725868 | 0.648741 | 1 |
| CA4-body (rh) | Gaussian | 1.90637E-05 | 4.21406E-06 | 4.523831 | 8.90427E-05 | 0.290117 | 0.018966 |
| fimbria (rh) | Gaussian | -1.36588E-05 | 3.79656E-06 | -3.59769 | 0.001138196 | 0.491817 | 0.242436 |
| CA3-head (rh) | Gaussian | 2.00883E-05 | 5.5117E-06 | 3.644674 | 0.001003458 | 0.654903 | 0.213736 |
| HATA (rh) | Gaussian | 6.09507E-06 | 3.06836E-06 | 1.986424 | 0.056187879 | 0.977153 | 1 |
| Whole_hippocampal_body (rh) | Gaussian | 0.000108168 | 3.00972E-05 | 3.593935 | 0.001149691 | 0.856462 | 0.244884 |
| Whole_hippocampal_head (rh) | Gaussian | 7.91138E-05 | 5.87471E-05 | 1.346684 | 0.188168589 | 0.327029 | 1 |
| Whole_hippocampus (rh) | Gaussian | 0.000298276 | 9.80602E-05 | 3.041762 | 0.004851089 | 0.846606 | 1 |
| AV (lh) | Gaussian | -7.4431E-06 | 4.16391E-06 | -1.78753 | 0.083959648 | 0.402397 | 1 |
| CeM (lh) | Gaussian | 2.2761E-06 | 3.67117E-06 | 0.619992 | 0.539943999 | 0.437212 | 1 |
| CL (lh) | Gaussian | -7.07401E-06 | 2.3461E-06 | -3.01523 | 0.005187239 | 0.015234 | 1 |
| CM (lh) | Gaussian | -1.73815E-05 | 8.03153E-06 | -2.16416 | 0.038539711 | 0.888815 | 1 |
| LD (lh) | Gaussian | -1.94366E-06 | 2.08958E-06 | -0.93017 | 0.359708591 | 0.037652 | 1 |
| LGN (lh) | Gaussian | -1.55075E-05 | 1.31142E-05 | -1.1825 | 0.246294729 | 0.010269 | 1 |
| LP (lh) | Gaussian | -2.00209E-05 | 5.04218E-06 | -3.97069 | 0.000413851 | 0.941216 | 0.08815 |
| L-Sg (lh) | Gaussian | -4.41278E-06 | 2.29565E-06 | -1.92224 | 0.064117752 | 0.682986 | 1 |
| MDl (lh) | Gaussian | 7.54059E-06 | 7.40858E-06 | 1.017819 | 0.316902694 | 0.023213 | 1 |
| MDm (lh) | Gaussian | 6.22124E-05 | 1.66233E-05 | 3.742476 | 0.000770864 | 0.255476 | 0.164194 |
| MGN (lh) | Gaussian | -2.75669E-06 | 7.68885E-06 | -0.35853 | 0.722456464 | 0.76616 | 1 |
| MV(Re) (lh) | Gaussian | 8.55924E-07 | 1.37625E-06 | 0.621927 | 0.538688393 | 0.272885 | 1 |
| Pc (lh) | Gaussian | 2.25391E-07 | 1.22695E-07 | 1.836997 | 0.076135661 | 0.78423 | 1 |
| Pf (lh) | Gaussian | -4.5994E-06 | 2.00487E-06 | -2.29412 | 0.028959131 | 0.84739 | 1 |
| Pt (lh) | Gaussian | -7.14437E-07 | 1.80067E-07 | -3.96762 | 0.000417349 | 0.368657 | 0.088895 |
| PuA (lh) | Gaussian | 5.48608E-06 | 7.03857E-06 | 0.779431 | 0.441831644 | 0.480375 | 1 |
| PuI (lh) | Gaussian | -2.22622E-05 | 1.93969E-05 | -1.14772 | 0.260154649 | 0.026573 | 1 |
| PuL (lh) | Gaussian | -1.33855E-05 | 1.40413E-05 | -0.9533 | 0.348058385 | 0.115361 | 1 |
| PuM (lh) | Gaussian | -2.07886E-05 | 4.87655E-05 | -0.4263 | 0.672934689 | 0.912932 | 1 |
| VA (lh) | Gaussian | 1.62195E-05 | 1.19194E-05 | 1.360765 | 0.183724055 | 0.735948 | 1 |
| VAmc (lh) | Gaussian | -9.12285E-08 | 9.92449E-07 | -0.09192 | 0.927370592 | 0.597398 | 1 |
| VLa (lh) | Gaussian | 1.80585E-06 | 1.53736E-05 | 0.117464 | 0.907275076 | 0.061149 | 1 |
| VLp (lh) | Gaussian | -2.25946E-05 | 2.14439E-05 | -1.05366 | 0.300449394 | 0.151598 | 1 |
| VM (lh) | Gaussian | -1.49159E-06 | 6.51748E-07 | -2.2886 | 0.029317876 | 0.925005 | 1 |
| VPL (lh) | Gaussian | -4.27435E-05 | 2.17532E-05 | -1.96493 | 0.058742382 | 0.15152 | 1 |
| Whole_thalamus (lh) | Gaussian | -0.000108589 | 0.000147529 | -0.73605 | 0.467414832 | 0.515381 | 1 |
| AV (rh) | Gaussian | -1.62037E-05 | 5.18231E-06 | -3.12673 | 0.003908596 | 0.047257 | 0.832531 |
| CeM (rh) | Gaussian | -2.36844E-06 | 4.52841E-06 | -0.52302 | 0.604804119 | 0.769011 | 1 |
| CL (rh) | Gaussian | -9.9566E-06 | 2.53368E-06 | -3.92969 | 0.000463071 | 0.637029 | 0.098634 |
| CM (rh) | Gaussian | -8.94225E-06 | 7.66407E-06 | -1.16678 | 0.252492394 | 0.083794 | 1 |
| LD (rh) | Gaussian | -6.42998E-06 | 2.26821E-06 | -2.83483 | 0.008128504 | 0.922395 | 1 |
| LGN (rh) | Gaussian | -1.85136E-06 | 1.44408E-05 | -0.1282 | 0.898843237 | 0.108222 | 1 |
| LP (rh) | Gaussian | -1.70503E-05 | 4.60872E-06 | -3.69957 | 0.000865611 | 0.393188 | 0.184375 |
| L-Sg (rh) | Gaussian | -2.34456E-06 | 1.41164E-06 | -1.66088 | 0.10715595 | 0.319903 | 1 |
| MDl (rh) | Gaussian | -2.28765E-05 | 1.02912E-05 | -2.22292 | 0.033902793 | 0.007704 | 1 |
| MDm (rh) | Gaussian | -1.52417E-05 | 2.80619E-05 | -0.54314 | 0.591043504 | 0.021028 | 1 |
| MGN (rh) | Gaussian | 4.03354E-06 | 5.12297E-06 | 0.787345 | 0.437256747 | 0.388574 | 1 |
| MV(Re) (rh) | Gaussian | -5.51331E-07 | 1.74772E-06 | -0.31546 | 0.754599054 | 0.977372 | 1 |
| Pc (rh) | Gaussian | 2.77929E-07 | 1.62263E-07 | 1.712825 | 0.097063565 | 0.014114 | 1 |
| Pf (rh) | Gaussian | -2.51937E-06 | 1.66841E-06 | -1.51004 | 0.141493981 | 0.312127 | 1 |
| Pt (rh) | Gaussian | -7.15397E-07 | 2.31088E-07 | -3.09578 | 0.004229692 | 0.219915 | 0.900924 |
| PuA (rh) | Gaussian | 8.40736E-06 | 6.97702E-06 | 1.205008 | 0.237620051 | 0.218025 | 1 |
| PuI (rh) | Gaussian | 8.38444E-06 | 9.48247E-06 | 0.884204 | 0.383617711 | 0.6517 | 1 |
| PuL (rh) | Gaussian | -1.98024E-06 | 9.71663E-06 | -0.2038 | 0.839886977 | 0.24736 | 1 |
| PuM (rh) | Gaussian | 3.74039E-05 | 3.57859E-05 | 1.045211 | 0.30427465 | 0.874972 | 1 |
| VA (rh) | Gaussian | -2.92847E-05 | 1.14337E-05 | -2.56126 | 0.015698253 | 0.482332 | 1 |
| VAmc (rh) | Gaussian | -1.15617E-06 | 1.00595E-06 | -1.14933 | 0.259500647 | 0.767051 | 1 |
| VLa (rh) | Gaussian | -2.21554E-05 | 1.74049E-05 | -1.27294 | 0.212816381 | 0.067449 | 1 |
| VLp (rh) | Gaussian | -2.76621E-05 | 2.37073E-05 | -1.16682 | 0.252475816 | 0.029042 | 1 |
| VM (rh) | Gaussian | -1.44712E-07 | 6.11742E-07 | -0.23656 | 0.814608497 | 0.157769 | 1 |
| VPL (rh) | Gaussian | -3.03767E-06 | 2.25242E-05 | -0.13486 | 0.89362164 | 0.113531 | 1 |
| Whole_thalamus (rh) | Gaussian | -0.000133965 | 0.00015485 | -0.86513 | 0.393832906 | 0.040326 | 1 |
