# Supplementary material for: Local brain volume reductions in patients with non-lesional epilepsy on 7T MRI
Source: Neuroradiology. 2025 Nov 18;68(6):1439–52. doi: 10.1007/s00234-025-03843-3 (PMC13323624; doi:10.1007/s00234-025-03843-3)
Supplement: Supplementary file 3 — Supplementary Material 3 [file 234_2025_3843_MOESM3_ESM.docx]

**Supplementary Table 2**

|  | Left-sided Focus | | | Right-sided Focus | | |
| --- | --- | --- | --- | --- | --- | --- |
|  | External Skullstrip | | No External Skullstrip | External Skullstrip | | No External Skullstrip |
|  | Mann-Whitney-U | GLM_FDR | GLM_FDR | Mann-Whitney-U | GLM_FDR | GLM_FDR |
| Ipsi-Amygdala | 1 | 1 | 0.9388 | 0.001 | 0.0002 | <0.001 |
| ctx-Ipsi-entorhinal | 0.0104 | 0.0137 | 0.1185 | 0.0035 | 0.0041 | <0.001 |
| ctx-Ipsi-fusiform | 0.0257 | 0.0076 | 0.506 | 0.5856 | 0.5089 | 0.0022 |
| ctx-Ipsi-lateralorbitofrontal | 0.6301 | 0.7124 | 0.7256 | 0.001 | <0.001 | 0.0007 |
| ctx-Ipsi-medialorbitofrontal | 0.0052 | 0.0012 | 0.8036 | 0.0015 | 0.0002 | 0.0115 |
| ctx-Ipsi-middletemporal | 1 | 1 | 0.1914 | 1 | 1 | 0.0667 |
| ctx-Ipsi-parahippocampal | 1 | 1 | 0.6244 | 0.0101 | 0.0011 | 0.0007 |
| ctx-Ipsi-superiortemporal | 1 | 1 | 0.0692 | 0.0035 | 0.0014 | 0.0004 |
| Hippocampal_tail (Ipsi) | 1 | 1 | 0.3708 | 0.0101 | 0.0132 | 0.032 |
| subiculum-body (Ipsi) | 1 | 1 | 0.247 | 0.0421 | 0.0042 | 0.0016 |
| hippocampal-fissure (Ipsi) | 1 | 1 | 0.0835 | 0.0051 | <0.001 | 0.0042 |
| presubiculum-body (Ipsi) | 0.0336 | 0.0054 | 0.0963 | 0.0015 | 0.0004 | 0.1357 |
| parasubiculum (Ipsi) | 1 | 1 | 0.3929 | 1 | 1 | 0.381 |
| CL (Ipsi) | 0.0015 | 0.0894 | 0.2299 | 0.0421 | 0.0986 | 0.0042 |

Comparison of p-values obtained from statistical tests between 12 individuals with epilepsy and 17 healthy controls, analyzed with and without the application of external skull stripping.
