# Supplementary material for: Local brain volume reductions in patients with non-lesional epilepsy on 7T MRI
Source: Neuroradiology. 2025 Nov 18;68(6):1439–52. doi: 10.1007/s00234-025-03843-3 (PMC13323624; doi:10.1007/s00234-025-03843-3)
Supplement: Supplementary file 4 — Supplementary Material 4 [file 234_2025_3843_MOESM4_ESM.docx]

**Supplementary Table 3**

Mean (mm³), coefficient of variation (CV), and FDR-adjusted p-values from the General Linear Model (GLM) comparing raw volumetric measures obtained with FastSurfer.

|  | Epilepsy | | | | | Healthy Control | | | | |
| --- | --- | --- | --- | --- | --- | --- | --- | --- | --- | --- |
|  | No external skull strip | | External skull strip | |  | No external skull strip | | External skull strip | |  |
|  | Mean (mm^3^) | CV (%) | Mean (mm^3^) | CV (%) | p_FDR | Mean (mm^3^) | CV (%) | Mean (mm^3^) | CV (%) | p_FDR |
| BrainSeg | 1026166.999 | 12.776 | 1074409.209 | 11.833 | 0.696 | 969658.273 | 7.739 | 1046236.261 | 7.160 | 0.021 |
| BrainSegNotVent | 1004144.488 | 13.022 | 1052251.623 | 11.969 | 0.696 | 951851.779 | 7.964 | 1028181.455 | 7.412 | 0.022 |
| SupraTentorial | 909078.959 | 13.107 | 950792.552 | 12.216 | 0.696 | 868794.593 | 7.986 | 931959.059 | 7.389 | 0.039 |
| SupraTentorialNotVent | 887056.449 | 13.380 | 928634.966 | 12.383 | 0.696 | 850988.099 | 8.263 | 913904.252 | 7.677 | 0.041 |
| SubCortGray | 54621.316 | 9.121 | 57204.774 | 9.489 | 0.696 | 51107.013 | 7.827 | 55644.593 | 6.841 | 0.008 |
| Left-Cerebral-White-Matter | 208823.776 | 14.811 | 217261.539 | 14.396 | 0.762 | 211882.858 | 8.912 | 220325.389 | 8.567 | 0.390 |
| Left-Lateral-Ventricle | 8230.556 | 43.117 | 8225.572 | 43.671 | 0.997 | 6753.573 | 42.598 | 6779.333 | 42.209 | 0.987 |
| Left-Inf-Lat-Vent | 421.269 | 29.868 | 381.021 | 28.060 | 0.696 | 420.264 | 26.467 | 383.425 | 26.980 | 0.556 |
| Left-Cerebellum-White-Matter | 7727.258 | 40.074 | 12522.270 | 9.718 | 0.002 | 5210.912 | 56.035 | 11452.979 | 13.573 | <0,001 |
| Left-Cerebellum-Cortex | 50300.883 | 16.062 | 49920.602 | 12.235 | 0.956 | 44357.460 | 14.634 | 46211.626 | 10.901 | 0.593 |
| Left-Thalamus | 7513.932 | 10.085 | 7579.193 | 9.192 | 0.940 | 7325.052 | 7.292 | 7448.195 | 7.387 | 0.761 |
| Left-Caudate | 3600.848 | 8.927 | 3659.232 | 8.821 | 0.874 | 3548.551 | 10.751 | 3617.214 | 10.637 | 0.843 |
| Left-Putamen | 4953.647 | 11.311 | 5127.405 | 11.716 | 0.743 | 4879.488 | 8.739 | 5011.080 | 8.572 | 0.611 |
| Left-Pallidum | 1947.398 | 7.910 | 1928.735 | 8.625 | 0.940 | 2091.083 | 7.695 | 2074.177 | 7.243 | 0.943 |
| Left-Hippocampus | 3796.331 | 13.998 | 4088.495 | 12.290 | 0.663 | 3169.797 | 18.050 | 3760.194 | 8.843 | 0.004 |
| Left-Amygdala | 1110.185 | 26.651 | 1560.419 | 13.942 | 0.006 | 855.086 | 27.383 | 1374.398 | 7.411 | <0,001 |
| Left-Accumbens-area | 556.037 | 11.222 | 597.202 | 12.854 | 0.626 | 541.235 | 11.461 | 570.555 | 12.250 | 0.390 |
| Left-VentralDC | 3757.921 | 7.610 | 4034.412 | 7.953 | 0.215 | 3722.431 | 9.729 | 4077.111 | 8.723 | 0.024 |
| Left-choroid-plexus | 572.095 | 20.365 | 575.273 | 21.849 | 0.976 | 477.903 | 25.702 | 495.793 | 25.176 | 0.914 |
| Right-Cerebral-White-Matter | 210778.621 | 14.749 | 217981.285 | 14.366 | 0.814 | 205932.818 | 8.737 | 217676.168 | 8.544 | 0.171 |
| Right-Lateral-Ventricle | 8084.001 | 47.934 | 8097.330 | 47.755 | 0.997 | 6215.488 | 33.887 | 6202.412 | 34.011 | 0.987 |
| Right-Inf-Lat-Vent | 425.440 | 30.222 | 408.571 | 32.949 | 0.935 | 396.884 | 34.032 | 416.089 | 34.965 | 0.923 |
| Right-Cerebellum-White-Matter | 9903.529 | 19.158 | 12317.062 | 9.940 | 0.020 | 7897.684 | 29.884 | 11678.413 | 10.250 | <0,001 |
| Right-Cerebellum-Cortex | 49156.370 | 12.885 | 48856.723 | 11.601 | 0.956 | 43397.625 | 10.436 | 44934.184 | 10.688 | 0.579 |
| Right-Thalamus | 7058.769 | 10.011 | 7325.316 | 9.505 | 0.696 | 6972.509 | 6.650 | 7263.630 | 6.559 | 0.186 |
| Right-Caudate | 3725.994 | 14.911 | 3782.191 | 15.283 | 0.940 | 3793.291 | 9.666 | 3882.732 | 10.158 | 0.761 |
| Right-Putamen | 4875.284 | 12.824 | 5130.694 | 12.608 | 0.696 | 4170.739 | 13.165 | 4897.708 | 7.597 | <0,001 |
| Right-Pallidum | 1951.285 | 11.743 | 1837.794 | 11.045 | 0.682 | 1748.101 | 9.630 | 1728.744 | 8.735 | 0.930 |
| Right-Hippocampus | 3922.519 | 10.838 | 4084.175 | 10.728 | 0.696 | 3149.120 | 15.418 | 3707.553 | 8.583 | 0.002 |
| Right-Amygdala | 1482.631 | 18.648 | 1709.528 | 13.868 | 0.221 | 739.654 | 34.946 | 1356.173 | 10.009 | <0,001 |
| Right-Accumbens-area | 575.659 | 21.962 | 626.634 | 20.895 | 0.696 | 486.609 | 18.368 | 578.160 | 10.184 | 0.006 |
| Right-VentralDC | 3792.876 | 6.862 | 4133.347 | 7.962 | 0.111 | 3914.267 | 8.151 | 4296.969 | 8.384 | 0.010 |
| Right-choroid-plexus | 748.584 | 20.847 | 683.871 | 19.500 | 0.696 | 637.529 | 21.152 | 631.370 | 20.318 | 0.987 |
| WM-hypointensities | 1724.490 | 56.803 | 1382.211 | 29.873 | 0.696 | 1197.548 | 25.979 | 1165.469 | 24.666 | 0.943 |
| 3rd-Ventricle | 923.794 | 35.016 | 957.089 | 34.611 | 0.940 | 773.380 | 38.651 | 797.844 | 36.817 | 0.961 |
| 4th-Ventricle | 1480.119 | 24.617 | 1704.613 | 35.237 | 0.696 | 1185.011 | 30.334 | 1388.611 | 29.900 | 0.290 |
| Brain-Stem | 16744.773 | 10.325 | 17328.471 | 9.312 | 0.696 | 14206.622 | 12.549 | 16452.992 | 8.830 | 0.002 |
| CSF | 1136.651 | 21.403 | 1124.245 | 19.741 | 0.956 | 946.463 | 18.258 | 959.930 | 17.980 | 0.961 |
| ctx-lh-caudalanteriorcingulate | 2625.695 | 18.443 | 2857.122 | 17.286 | 0.696 | 2799.503 | 17.274 | 2989.003 | 14.987 | 0.442 |
| ctx-lh-caudalmiddlefrontal | 6545.739 | 16.668 | 6311.088 | 14.911 | 0.814 | 7211.875 | 15.895 | 6921.755 | 14.544 | 0.685 |
| ctx-lh-cuneus | 4216.905 | 13.095 | 4378.203 | 13.746 | 0.762 | 4301.306 | 17.490 | 4288.940 | 18.604 | 0.987 |
| ctx-lh-entorhinal | 860.993 | 40.116 | 1501.676 | 15.204 | 0.001 | 368.546 | 53.048 | 1038.573 | 16.416 | <0,001 |
| ctx-lh-fusiform | 5309.686 | 31.407 | 6966.358 | 17.799 | 0.114 | 4034.476 | 21.173 | 5471.570 | 16.759 | <0,001 |
| ctx-lh-inferiorparietal | 11687.747 | 21.448 | 10909.681 | 19.534 | 0.696 | 11460.401 | 12.888 | 10584.648 | 10.325 | 0.153 |
| ctx-lh-inferiortemporal | 7686.777 | 44.261 | 10183.707 | 16.681 | 0.215 | 4409.154 | 34.186 | 7988.559 | 16.399 | <0,001 |
| ctx-lh-isthmuscingulate | 2359.574 | 13.757 | 2426.935 | 13.849 | 0.849 | 2252.531 | 16.076 | 2250.472 | 16.365 | 0.987 |
| ctx-lh-lateraloccipital | 10728.034 | 14.572 | 9670.463 | 13.427 | 0.393 | 10993.222 | 15.193 | 9669.454 | 12.901 | 0.041 |
| ctx-lh-lateralorbitofrontal | 6462.297 | 32.750 | 8750.853 | 12.017 | 0.040 | 4763.479 | 34.254 | 7748.020 | 10.517 | <0,001 |
| ctx-lh-lingual | 6167.084 | 18.093 | 6608.490 | 17.182 | 0.696 | 5792.576 | 15.268 | 5954.131 | 14.907 | 0.843 |
| ctx-lh-medialorbitofrontal | 2208.891 | 35.380 | 3952.234 | 15.867 | <0,001 | 1824.010 | 25.884 | 3015.660 | 11.946 | <0,001 |
| ctx-lh-middletemporal | 11687.362 | 24.195 | 12553.795 | 17.199 | 0.696 | 9178.058 | 21.819 | 11828.656 | 11.978 | <0,001 |
| ctx-lh-parahippocampal | 1294.224 | 19.498 | 1772.140 | 14.284 | 0.003 | 1036.830 | 25.615 | 1421.818 | 17.511 | <0,001 |
| ctx-lh-paracentral | 3523.542 | 14.424 | 3717.154 | 14.127 | 0.696 | 4088.712 | 17.032 | 4143.388 | 15.043 | 0.961 |
| ctx-lh-parsopercularis | 3822.654 | 16.689 | 3978.593 | 12.868 | 0.762 | 4026.846 | 15.335 | 4164.113 | 14.019 | 0.761 |
| ctx-lh-parsorbitalis | 1732.826 | 30.196 | 2109.722 | 13.425 | 0.215 | 1386.389 | 45.189 | 2217.143 | 15.643 | <0,001 |
| ctx-lh-parstriangularis | 4015.014 | 19.417 | 4367.145 | 22.446 | 0.696 | 4190.255 | 17.178 | 4506.545 | 18.590 | 0.442 |
| ctx-lh-pericalcarine | 1865.158 | 14.169 | 1912.356 | 14.048 | 0.874 | 2092.472 | 22.488 | 2105.698 | 20.306 | 0.987 |
| ctx-lh-postcentral | 9688.863 | 10.429 | 9282.744 | 10.376 | 0.696 | 9749.118 | 13.036 | 9585.119 | 13.254 | 0.930 |
| ctx-lh-posteriorcingulate | 2955.025 | 17.254 | 3036.296 | 19.498 | 0.905 | 3149.556 | 13.356 | 3153.681 | 13.373 | 0.987 |
| ctx-lh-precentral | 12197.163 | 11.290 | 11785.575 | 9.637 | 0.696 | 12421.246 | 8.482 | 12455.105 | 7.774 | 0.987 |
| ctx-lh-precuneus | 8578.404 | 17.277 | 8809.363 | 17.501 | 0.905 | 8655.845 | 11.352 | 8679.488 | 11.181 | 0.987 |
| ctx-lh-rostralanteriorcingulate | 2238.016 | 28.604 | 3147.079 | 25.703 | 0.072 | 2322.890 | 17.937 | 2919.012 | 15.323 | 0.002 |
| ctx-lh-rostralmiddlefrontal | 10994.499 | 16.114 | 10880.604 | 16.624 | 0.956 | 11099.104 | 18.091 | 11995.687 | 15.381 | 0.378 |
| ctx-lh-superiorfrontal | 22358.744 | 13.517 | 23344.792 | 11.579 | 0.696 | 23385.014 | 12.994 | 24244.614 | 12.574 | 0.662 |
| ctx-lh-superiorparietal | 9615.056 | 17.271 | 9305.755 | 15.156 | 0.849 | 9956.019 | 13.466 | 9412.139 | 12.078 | 0.394 |
| ctx-lh-superiortemporal | 14407.526 | 20.815 | 16344.045 | 12.558 | 0.379 | 10571.528 | 16.733 | 14063.927 | 9.048 | <0,001 |
| ctx-lh-supramarginal | 10313.529 | 16.129 | 8839.380 | 13.681 | 0.186 | 10290.586 | 14.335 | 9029.938 | 14.390 | 0.040 |
| ctx-lh-transversetemporal | 1027.080 | 17.502 | 1009.646 | 17.671 | 0.940 | 958.530 | 16.694 | 994.779 | 17.915 | 0.785 |
| ctx-lh-insula | 5373.521 | 15.442 | 5831.598 | 14.357 | 0.667 | 5014.292 | 11.988 | 5285.268 | 11.561 | 0.390 |
| ctx-rh-caudalanteriorcingulate | 1847.814 | 23.973 | 2017.697 | 25.289 | 0.696 | 1848.960 | 17.684 | 1978.187 | 19.250 | 0.518 |
| ctx-rh-caudalmiddlefrontal | 6131.252 | 21.884 | 6162.156 | 20.457 | 0.976 | 6593.413 | 15.782 | 6578.712 | 14.899 | 0.987 |
| ctx-rh-cuneus | 3794.889 | 10.730 | 3900.190 | 11.668 | 0.807 | 3924.735 | 15.537 | 3884.710 | 16.278 | 0.985 |
| ctx-rh-entorhinal | 942.341 | 29.536 | 1479.406 | 18.303 | 0.003 | 165.004 | 78.843 | 974.724 | 30.702 | <0,001 |
| ctx-rh-fusiform | 6144.063 | 17.205 | 7095.136 | 14.646 | 0.215 | 4679.768 | 14.821 | 5865.505 | 12.071 | <0,001 |
| ctx-rh-inferiorparietal | 13855.533 | 16.970 | 12443.845 | 15.850 | 0.505 | 13317.682 | 12.139 | 12459.652 | 11.903 | 0.255 |
| ctx-rh-inferiortemporal | 8605.630 | 23.406 | 10149.725 | 13.233 | 0.215 | 5113.885 | 27.439 | 8974.161 | 12.348 | <0,001 |
| ctx-rh-isthmuscingulate | 2106.986 | 14.735 | 2140.160 | 15.743 | 0.940 | 2162.563 | 14.985 | 2123.093 | 15.420 | 0.930 |
| ctx-rh-lateraloccipital | 11625.089 | 10.939 | 10944.796 | 11.912 | 0.682 | 10846.892 | 14.148 | 9973.117 | 12.128 | 0.177 |
| ctx-rh-lateralorbitofrontal | 5842.527 | 24.286 | 8245.169 | 13.917 | 0.004 | 3269.704 | 33.351 | 6056.866 | 9.148 | <0,001 |
| ctx-rh-lingual | 6779.934 | 11.914 | 7022.429 | 12.573 | 0.759 | 6331.229 | 13.211 | 6346.104 | 13.273 | 0.987 |
| ctx-rh-medialorbitofrontal | 2144.548 | 30.096 | 3833.527 | 11.302 | <0,001 | 1396.632 | 30.682 | 2790.708 | 14.036 | <0,001 |
| ctx-rh-middletemporal | 12251.488 | 19.865 | 12717.477 | 17.680 | 0.849 | 9545.371 | 19.189 | 12182.956 | 9.575 | <0,001 |
| ctx-rh-parahippocampal | 1679.862 | 19.518 | 1792.131 | 16.054 | 0.696 | 1109.720 | 16.957 | 1335.012 | 13.866 | 0.006 |
| ctx-rh-paracentral | 3556.878 | 11.175 | 3722.250 | 10.563 | 0.696 | 4080.885 | 12.221 | 4126.296 | 11.355 | 0.961 |
| ctx-rh-parsopercularis | 3799.844 | 17.656 | 4265.411 | 15.334 | 0.437 | 3492.243 | 16.740 | 4089.097 | 10.184 | 0.007 |
| ctx-rh-parsorbitalis | 2111.356 | 19.412 | 2474.025 | 13.208 | 0.186 | 894.292 | 70.400 | 2086.022 | 14.222 | <0,001 |
| ctx-rh-parstriangularis | 3779.993 | 16.280 | 3900.812 | 20.332 | 0.878 | 3523.484 | 29.245 | 4171.064 | 19.913 | 0.141 |
| ctx-rh-pericalcarine | 2084.038 | 13.547 | 2105.515 | 13.299 | 0.955 | 2456.944 | 23.690 | 2467.775 | 22.534 | 0.987 |
| ctx-rh-postcentral | 9039.115 | 6.458 | 8819.333 | 8.293 | 0.696 | 8870.538 | 13.561 | 8665.651 | 11.719 | 0.843 |
| ctx-rh-posteriorcingulate | 3105.940 | 11.896 | 3259.547 | 13.559 | 0.696 | 3113.492 | 17.477 | 3154.855 | 16.337 | 0.961 |
| ctx-rh-precentral | 11001.838 | 11.624 | 10971.172 | 11.457 | 0.976 | 11378.380 | 9.946 | 11554.332 | 9.584 | 0.891 |
| ctx-rh-precuneus | 9608.150 | 19.612 | 9706.978 | 19.552 | 0.956 | 9620.404 | 12.148 | 9585.484 | 11.877 | 0.987 |
| ctx-rh-rostralanteriorcingulate | 1601.156 | 26.712 | 2051.173 | 23.378 | 0.186 | 1281.723 | 32.268 | 1635.877 | 24.207 | 0.046 |
| ctx-rh-rostralmiddlefrontal | 10192.382 | 16.411 | 11032.529 | 16.149 | 0.696 | 10509.730 | 18.078 | 12424.693 | 15.358 | 0.022 |
| ctx-rh-superiorfrontal | 24153.922 | 15.987 | 26070.067 | 14.318 | 0.696 | 24343.478 | 11.735 | 26246.252 | 11.226 | 0.164 |
| ctx-rh-superiorparietal | 10234.570 | 18.311 | 9588.647 | 21.472 | 0.696 | 10457.609 | 13.444 | 9851.862 | 12.238 | 0.378 |
| ctx-rh-superiortemporal | 12833.757 | 20.726 | 15128.023 | 12.731 | 0.186 | 8989.924 | 21.682 | 13058.280 | 10.066 | <0,001 |
| ctx-rh-supramarginal | 9509.708 | 16.604 | 8514.280 | 15.257 | 0.447 | 9009.688 | 11.986 | 8345.025 | 11.385 | 0.164 |
| ctx-rh-transversetemporal | 825.440 | 18.514 | 838.369 | 16.381 | 0.940 | 681.073 | 14.745 | 745.494 | 16.117 | 0.224 |
| ctx-rh-insula | 5370.574 | 16.976 | 5868.586 | 15.535 | 0.667 | 4074.048 | 17.104 | 5234.164 | 11.533 | <0,001 |
